# Blood DNA methylation signature of diet quality and association with cardiometabolic traits

**DOI:** 10.1093/eurjpc/zwad317

**Published:** 2023-10-04

**Authors:** Jorge Domínguez-Barragán, Alba Fernández-Sanlés, Álvaro Hernáez, Joana Llauradó-Pont, Jaume Marrugat, Oliver Robinson, Ioanna Tzoulaki, Roberto Elosua, Camille Lassale

**Affiliations:** Hospital del Mar Research Institute (IMIM), Programme of Epidemiology and Public Health, Dr Aiguader, 88, 08003 Barcelona, Spain; MRC Unit for Lifelong Health and Ageing, University College London, London WC1E 7HB, UK; Population Health Sciences, Bristol Medical School, University of Bristol, Bristol BS8 2BN, UK; Centre for Fertility and Health, Norwegian Institute of Public Health, Oslo 0463, Norway; Blanquerna School of Health Sciences, Universitat Ramon Llull, 08025 Barcelona, Spain; Consortium for Biomedical Research—Pathophysiology of Obesity and Nutrition (CIBEROBN), Instituto de Salud Carlos III, Monforte de Lemos 3-5, 08029 Madrid, Spain; Barcelona Institute of Global Health (ISGlobal), Dr Aiguader 88, 08003, Barcelona, Spain; Hospital del Mar Research Institute (IMIM), Programme of Epidemiology and Public Health, Dr Aiguader, 88, 08003 Barcelona, Spain; Consortium for Biomedical Research—Cardiovascular Diseases (CIBERCV), Instituto de Salud Carlos III, Madrid, Spain; μedical Research Council Centre for Environment and Health, School of Public Health, Imperial College London, London W2 1PG, UK; Centre for Systems Biology, Biomedical Research Foundation of the Academy of Athens, 115 27 Athens, Greece; Department of Epidemiology and Biostatistics, School of Public Health, Imperial College London, London W2 1PG, UK; Hospital del Mar Research Institute (IMIM), Programme of Epidemiology and Public Health, Dr Aiguader, 88, 08003 Barcelona, Spain; Consortium for Biomedical Research—Cardiovascular Diseases (CIBERCV), Instituto de Salud Carlos III, Madrid, Spain; Faculty of Medicine, University of Vic—Central University of Catalunya, Ctra. de Roda, 70, 08500 Vic, Spain; Hospital del Mar Research Institute (IMIM), Programme of Epidemiology and Public Health, Dr Aiguader, 88, 08003 Barcelona, Spain; Consortium for Biomedical Research—Pathophysiology of Obesity and Nutrition (CIBEROBN), Instituto de Salud Carlos III, Monforte de Lemos 3-5, 08029 Madrid, Spain; Barcelona Institute of Global Health (ISGlobal), Dr Aiguader 88, 08003, Barcelona, Spain; Universitat Pompeu Fabra (UPF), Dr Aiguader 88, 08003, Barcelona, Spain

**Keywords:** DNA methylation, Diet quality, Cardiovascular disease, Epidemiology, Nutrition

## Abstract

**Aims:**

Diet quality might influence cardiometabolic health through epigenetic changes, but this has been little investigated in adults. Our aims were to identify cytosine–phosphate–guanine (CpG) dinucleotides associated with diet quality by conducting an epigenome-wide association study (EWAS) based on blood DNA methylation (DNAm) and to assess how diet-related CpGs associate with inherited susceptibility to cardiometabolic traits: body mass index (BMI), systolic blood pressure (SBP), triglycerides, type 2 diabetes (T2D), and coronary heart disease (CHD).

**Methods and results:**

Meta-EWAS including 5274 participants in four cohorts from Spain, the USA, and the UK. We derived three dietary scores (exposures) to measure adherence to a Mediterranean diet, to a healthy plant-based diet, and to the Dietary Approaches to Stop Hypertension. Blood DNAm (outcome) was assessed with the Infinium arrays Human Methylation 450K BeadChip and MethylationEPIC BeadChip. For each diet score, we performed linear EWAS adjusted for age, sex, blood cells, smoking and technical variables, and BMI in a second set of models. We also conducted Mendelian randomization analyses to assess the potential causal relationship between diet-related CpGs and cardiometabolic traits. We found 18 differentially methylated CpGs associated with dietary scores (*P* < 1.08 × 10^−7^; Bonferroni correction), of which 12 were previously associated with cardiometabolic traits. Enrichment analysis revealed overrepresentation of diet-associated genes in pathways involved in inflammation and cardiovascular disease. Mendelian randomization analyses suggested that genetically determined methylation levels corresponding to lower diet quality at cg02079413 (*SNORA54*), cg02107842 (*MAST4*), and cg23761815 (*SLC29A3*) were causally associated with higher BMI and at cg05399785 (*WDR8*) with greater SBP, and methylation levels associated with higher diet quality at cg00711496 (*PRMT1*) with lower BMI, T2D risk, and CHD risk and at cg0557921 (AHRR) with lower CHD risk.

**Conclusion:**

Diet quality in adults was related to differential methylation in blood at 18 CpGs, some of which related to cardiometabolic health.

## Graphical Abstract

**Graphical Abstract:**
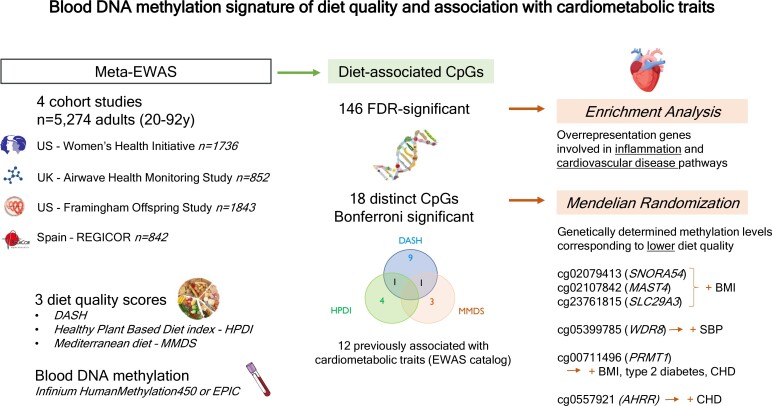

**See the editorial comment for this article ‘The epigenome, the missing link between diet and cardiovascular disease?’, by P. Marques-Vidal, https://doi.org/10.1093/eurjpc/zwad324.**

## Introduction

Cardiovascular diseases are the leading causes of death and morbidity globally, and they result from a complex interaction between genetics, lifestyle, and environmental factors.^1^ The epigenome, which controls the differential expression of genes in each cell type, is both heritable and modifiable by lifestyle and environmental factors^2^ and has the potential to represent gene–lifestyle/environment interactions. One key epigenetic mechanism is DNA methylation (DNAm), the reversible addition of a methyl group at the five-carbon position of a cytosine, mainly in cytosine–phosphate–guanine (CpG) dinucleotide sites in humans.

Poor quality of diet is a key cardiovascular risk factor.^3,4^ As foods are a mixture of complex nutrients rather than isolated elements, describing dietary patterns has received increasing interest in nutritional epidemiology.^5^ Some dietary components (e.g. folate, vitamins B6 and B12, betaine, methionine, choline, polyphenols, flavonoids, and polyunsaturated fatty acids) are key determinants of DNAm levels,^6^ which in turn could mediate the effects of diet on health-related outcomes.^7^ Maternal diet composition has consequences on foetal programming, growth, and metabolism,^6^ but the effect of diet on epigenetic markers has been less studied in adults. Recent epigenome-wide association studies (EWASs) started to uncover differentially methylated CpGs in relation to diet. For example, 11 CpGs were found to be associated with coffee consumption.^8^ Two previous EWASs measured overall diet quality and found association of CpG methylation levels with dietary patterns. One study^9^ found 30 distinct CpGs to be associated to the Modified Mediterranean Diet Score (MMDS) only (6 CpGs), the Alternative Health Eating Index (AHEI) (20 CpGs), or both diet scores (4 CpGs). The other study found 24 additional CpGs associated with the AHEI.^10^ Some of these CpGs associated with diet quality were linked to cardiometabolic health outcomes such as obesity, diabetes, dyslipidaemia and elevated blood pressure, and all-cause mortality,^9^ therefore might point to an epigenetic mechanism underlying the association between diet quality and cardiometabolic health. If these CpGs are related to differences in cardiometabolic disease risk using approaches less affected by residual confounding, such as Mendelian randomization (MR, which also suggests potential causal associations),^11^ this would indicate a possible mediating effect of methylation on the preventive role of diet in these diseases.

Using data from four cohorts with information on blood DNAm, we aimed to uncover differentially methylated CpGs associated with three diet quality scores [MMDS, healthy plant-based diet (HPDI), and Dietary Approaches to Stop Hypertension (DASH)]. We also aimed to test whether there was a causal relationship between diet-associated CpGs and cardiometabolic factors [body mass index (BMI), diabetes, blood lipids, and blood pressure] by using MR analyses.

## Methods

### Study design and populations

**Figure 1:**
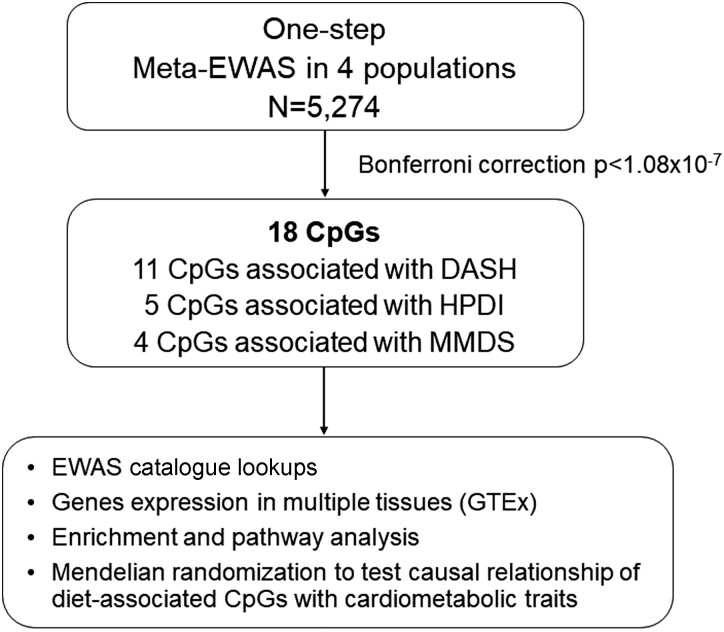
Study design of the one-step meta-epigenome-wide association between three diet quality scores and DNA methylation in four adult cohorts (REGICOR, Airwave, Framingham Offspring Study, and Women’s Health initiative). Abbreviations: meta-EWAS, meta-analysis of epigenome-wide association studies; CpG, cytosine–phosphate–guanine; DASH, Dietary Approaches to Stop Hypertension; MMDS, Modified Mediterranean Diet Score; HPDI, Healthful Plant-based Diet Index.

We designed a meta-EWAS (*Figure 1*) using four independent populations, predominantly of European ancestry: REgistre Gironí del COR (REGICOR, *n* = 842), a population-based cohort of adults aged 35–79 years old (average age 57.3 years; 50.4% female) living in the Girona province, Spain; the Framingham Offspring Study (FOS, *n* = 1843), a population of adults in the USA, aged 40–92 years old (average age 66.4 years; 56.7% female)^12^; the Women’s Health Initiative (WHI, *n* = 1736), a population of US postmenopausal women aged 50–79 years old (average age 64.2 years)^13^; and the Airwave Health Monitoring Study (AIRWAVE, *n* = 852) of police officers and staff in Great Britain, a population aged 20–64 years old (average age 41.1 years; 42.9% female).^14^ Both FOS and WHI data were available in the database of Genotypes and Phenotypes (http://dbgap.ncbi.nlm.nih.gov; Project #9047). The AIRWAVE data were available after approval by Dementia Platform UK (Study #0438) on https://portal.dementiasplatform.uk/. Participants in all four studies gave their informed written consent. The FHS, WHI, and AIRWAVE studies were approved by ethics committee. The REGICOR study and ancillary protocols were approved by the Parc de Salut Mar Ethics Committee (2012/4729/I; 2015/6199/I; and 2018/7855/I)

### Diet assessment and diet quality scores

Dietary data were obtained from semi-quantitative food frequency questionnaires in REGICOR,^15^ FOS,^16^ and WHI^17^ participants. In AIRWAVE, these data were obtained from 7-day food diaries.^18^ Implausible questionnaires and energy under- and overreporters were excluded as described in Supplementary material online, *Text S1*.

Diet quality was assessed using three different diet scores: MMDS,^9^ HPDI,^19^ and DASH.^20^ These three scores were chosen because they mostly rely on food group intake and include only minimal micronutrient intake estimation, which are less reliable with food frequency questionnaires. Moreover, these scores have been associated with a lower BMI and other cardiometabolic health outcomes in many studies.^19,21–23^ Their computation is detailed in Supplementary material online, *Text S2*. Briefly, the MMDS includes nine components: vegetables, fruits, nuts, legumes, whole grains, fish, ratio of monounsaturated fatty acids to saturated fatty acids (positive), red and processed meats (negative), and alcohol (moderate intake). The DASH diet score includes eight components: fruits, vegetables, nuts and legumes, low-fat dairy products, whole grains (positive), sodium, sweetened beverages, and red and processed meats (negative). The HPDI includes 18 components: seven healthy plant foods (whole grains, fruits, vegetables, nuts, legumes, vegetable oils, and tea and coffee), five less healthy plant foods (fruit juices, refined grains, potatoes, sugar-sweetened beverages, and sweets and desserts), and six animal foods (animal fat including butter or lard, dairy, eggs, fish and seafood, meat, and miscellaneous animal-based foods). The three scores reflect widely used dietary recommendations but yet are different in their computation and content (e.g. low-fat dairy considered a healthy item in the DASH diet, whereas dairy is a detrimental item in HPDI and is absent from MMDS calculation). We calculated Pearson’s correlation coefficients between the three diet scores, in each cohort, to assess the similarities between them. We also calculated Pearson’s correlation coefficients between EWAS coefficients of the three diet scores to assess consistency or differences in direction of the association between CpG methylation and each diet score.

### DNA methylation

DNA extraction and methylation assessment methods were previously described for all four cohorts.^12,24,25^ Briefly, DNA was extracted from peripheral blood cells in all cohorts (whole blood or buffy coat). DNAm levels were measured using the Illumina HumanMethylation450 BeadChip (450K) array in REGICOR-450K (*n* = 573), WHI, and FOS and Illumina MethylationEPIC BeadChip (EPIC) in REGICOR-EPIC (*n* = 269) and AIRWAVE. The 450K array analyses over 485 000 CpGs per sample, and EPIC analyses over 850 000 CpGs, including >90% of 450K CpGs.^26^ We focused on autosomal CpGs available in the 450K array (i.e. the EPIC-specific CpGs were excluded). We obtained genomic information of the CpG using the manifest and annotation provided by Illumina and contained in the corresponding R packages available through Bioconductor (assembly reference hg19).

For the measurement of methylation levels, *M*-values of each CpG were used. Although methylation can be measured also as a *β*-value, *M*-values are more statistically robust^27^ and are calculated as follows:


$$M_{\mathrm{value}}=\mathrm{lo}g_{2}\frac{\left(\mathrm{Mi}+\alpha \right)}{\left(\mathrm{Ui}+\alpha \right)}$$

with Mi = intensity of methylated probes; Ui = intensity of unmethylated probes; and *α* = 1 (constant offset). CpGs with *M*-values close to 0 are half-methylated. Positive values indicate the presence of more methylated than unmethylated cytosines, while negative *M*-values denote the opposite ratio. *M*-values were normalized with the *dasen* function from the *wateRmelon* R package^28^ and standardized by batch as previously reported.^12^

Quality control of the raw DNAm data was previously described for the REGICOR, WHI, and FOS samples by our group.^12,24^ For AIRWAVE, quality control is described in Supplementary material online, *Text S3*. A total of 463 932 CpGs were finally included in the analysis. Supplementary material online, *Figure S1* shows a Venn diagram of the number of CpGs available in each sample.

### Covariates

Age, sex, ethnicity (AIRWAVE and WHI), and smoking status (never, ex-smoker, and current smoker) were collected by questionnaires, and BMI was measured by trained nurses in all cohorts. In AIRWAVE and WHI, covariates were collected at the same visit as diet quality data and blood samples. In REGICOR, covariates and blood samples were collected at follow-up, 6 years later than diet quality (assessed at baseline). In FOS, covariates and blood samples were collected at Exam 8, 7 years later than diet quality (collected at Exam 7). Family relationship was available in FOS, allowing to create a kinship variable used as a random effect in regression models. Peripheral blood cell counts (NK, CD8T, CD4T, B cells, monocytes, and granulocytes) were estimated using the Houseman algorithm^29^ with the *minfi* R package^30^ integrating data from the *FlowSorted.Blood.450 k* R package.^31^

In order to remove noise from potential and non-measured sources of variation due to technical and biological confounders, surrogate variables were estimated for REGICOR (two variables for DASH, HPDI, and MMDS for 450k array; three variables for DASH, six variables for HPDI, and seven variables for MMDS for epic array), FOS (two variables for DASH, MMDS, and HPDI), and WHI (six variables for DASH, two variables for MMDS and HPDI) using the *sva* R package^32^ to account for unknown sources of potential technical or biological confounding. In AIRWAVE, due to computational limitations, we did not calculate them but included the chip as a random effect in analyses to account for batch effect.

If EWAS results were inflated (lambda values > 1.05), we corrected the coefficients, the standard errors, and the *P*-values using the *bacon* R package.^33^ Q–Q plots of *P*-value distribution (observed vs. expected) and Manhattan plots before and after correction (when needed) are shown in Supplementary material online, *Figures S2* and *S3*.

### Meta-epigenome-wide association study of diet quality scores

All diet scores were standardized in each cohort and used as continuous exposure variable. We performed EWAS for each of the three diet scores, in all samples, using DNAm *M*-values as the outcome variable. We used linear models in WHI and REGICOR populations and linear mixed models with random effects in FOS and AIRWAVE. We defined two models based on the covariates we adjusted for. Model 1 was adjusted for blood cell count, age (years), total energy intake (kcal), sex (except in WHI, where all participants are women), smoking status (former, current, or never smokers), and surrogate variables (except in AIRWAVE). In WHI, we further adjusted for ethnicity [White *n* = 841 (48%) and non-White]. In AIRWAVE, due to the very low number (*n* = 29) of non-White participants, they were excluded from the analysis (Supplementary material online, *Text S2*). Model 2 was further adjusted for BMI (kg/m^2^). To assess if the associations were not merely explained by adiposity, we calculated the attenuation (in %) of the coefficients between Model 1 and Model 2 (that includes BMI) using the following formula:


$$\mathrm{Attenuation}=\frac{\mathrm{Coefficientmodel}1\text{-}\mathrm{Coefficientmodel}2}{\mathrm{Coefficientmodel}1}\times 100$$

We considered an attenuation of 10% as an important contribution of BMI to explain the association. In the linear mixed models in FOS, we used the family relationship variable as a grouping factor (random effect), and in AIRWAVE, we used the chip information from EPIC arrays to account for batch effect.

For each diet score, we meta-analysed the results from the five samples using the R package *meta*.^34^ We report significant fixed effects, except in the presence of substantial heterogeneity between cohorts, defined as a value of *I*^2^ > 50% or a *Q*-test < 0.05, where we report the random effects. To define the statistical *P*-value threshold, we applied the Bonferroni correction for multiple testing,^35^ corresponding to a *P* < 1.08 × 10^−7^, and reported those CpGs with *P*-value lower than the threshold as main results. We also identified those CpGs with a false discovery rate (FDR)–corrected *P* < 0.05 using the Benjamini–Hochberg method.^36^

Due to the ethnic diversity of the WHI sample, we conducted a sensitivity analysis excluding this sample from the meta-analysis to check if the results were influenced by ancestry.

### Functional interpretation of diet-related CpGs

We used the GTEx expression database (https://gtexportal.org/home/) to provide further insight in the differential expression of the identified genes across different human tissues. Furthermore, we looked for each CpG on the EWAS catalogue (http://www.ewascatalog.org/) to report any previously reported association with cardiometabolic health outcomes, lifestyle, or dietary intake.

To identify pathways and molecular functions of the diet-related CpG mapping to genes, we conducted functional enrichment analysis including all the FDR-significant CpGs to allow a sufficient number of genes to be included. We used the *missmethyl* R package,^37^ as implemented in the Functional Enrichment module of the EASIER R package.^38^ This is a method to identify pathways in which the genes annotated to the CpGs are overrepresented. We used public annotation data from gene ontology (GO), Kyoto Encyclopedia of Genes and Genomes (KEGG), the Molecular Signatures Database (MSigDB), and ConsensusPathDB (http://consensuspathdb.org) that includes four databases [protein complex–based sets (C), GO level 2 (G2), GO level 3 (G3), and manually curated pathways from pathway databases (P)]. Finally, we retrieved information related to the CpG location (gene position and island relative positions) and chromatin states using Roadmap blood 15 reference chromatin states.^39^

### Mendelian randomization

We studied the effects of the identified CpGs on inherited susceptibility to four cardiometabolic traits [BMI, triglycerides, systolic blood pressure (SBP), and type 2 diabetes (T2D)] and coronary heart disease (CHD) to explore whether those CpGs could play a role as mediators of the association between diet and cardiometabolic disorders. To test for causality, we performed two-sample MR analyses using the R package *TwoSampleMR.*^40^ Regarding the exposure, we searched for single nucleotide polymorphisms (SNPs) that are linked to the presence of the diet-associated CpGs in the GoDMC methylation quantitative trait loci (mQTL) database (http://mqtldb.godmc.org.uk/).^41^ We used a linkage disequilibrium filter of 0.8. Regarding the outcomes, we used the most updated genome-wide association study (GWAS) summary statistics for BMI,^42^ triglycerides,^43^ SBP,^44^ T2D,^45^ and CHD^46^ all of them based on populations of European ancestry. We took this decision because there were only a minority of non-White participants in the meta-EWAS data set (*n* = 895); therefore, 83% of our sample was from European ancestry. We looked for the SNPs linked to the identified CpGs in the summary statistics of the GWASs on the aforementioned cardiometabolic traits. We excluded genetic variants showing a stronger association with the outcome than with the exposure using the Steiger filtering (to avoid bias due to reverse causation^47^), and palindromic SNPs with allele frequencies close to 0.5, and we harmonized the format of both data sets. Finally, we used a multiplicative random effects inverse-variance weighted method as the main MR approach.^47^ We also studied the three assumptions that must be met to obtain valid conclusions in MR (a robust relationship between genetic instruments and the exposure, lack of confounding of the genetic instrument–outcome associations, and the exclusive link of genetic instruments to the outcome through the exposure of interest^47^) as reported in Supplementary material online, *Text S4*.

## Results

We analysed the association of diet and DNAm for a total of 5274 participants (67.7% women). All participants were from European ancestry except in WHI (52.9% of non-White participants) (*Table 1*).

**Table 1:** Descriptive characteristics of study populations

	WHI	FHS	REGICOR-450	REGICOR-EPIC	AIRWAVE
*n*	1736	1843	573	269	853
Age (years), mean (SD)	64.2 (7.05)	66.4 (8.87)	57.9 (11.44)	56.0 (7.03)	41.1 (9.37)
Female sex (*n*, %)	1736 (100)	1045 (56.7)	285 (49.7)	139 (51.7)	366 (42.9)
BMI (kg/m^2^), mean (SD)	29.8 (6.02)	28.2 (5.37)	27.2 (3.93)	28.3 (5.87)	27.1 (4.41)
Smoking status					
Never	938 (54.0)	1640 (89.0)	302 (52.7)	162 (60.2)	558 (65.4)
Ex-smoker	634 (36.5)	32.0 (1.70)	147 (25.7)	63.0 (23.4)	214 (25.1)
Smoker	164 (9.40)	171 (9.30)	124 (21.6)	44 (16.4)	81.0 (9.50)
Energy kcal/day [mean (SD)]	1628 (636)	1841 (573)	2319 (596)	2459 (593)	1925 (482)
Alcohol g/day [mean (SD)]	3.75 (10.7)	10.2 (14.8)	9.64 (14.0)	11.0 (14.0)	15.9 (17.1)
MMDS [mean (SD)]	12.0 (4.04)	12.1 (4.45)	11.8 (4.42)	11.9 (4.36)	11.8 (4.29)
DASH [mean (SD)]	24.3 (4.94)	24.4 (4.67)	24.5 (5.24)	23.9 (4.95)	23.9 (5.04)
HPDI [mean (SD)]	54.7 (7.34)	52.1 (6.71)	57.4 (6.97)	57.6 (7.00)	55.7 (7.35)

### Diet-associated CpGs

The diet scores were moderately correlated (see Supplementary material online, *Figure S4*). After meta-analysing the results from the individual populations with Model 1 (adjusted for cell count, age, sex, energy intake, smoking, and surrogates), we found a total of 18 CpGs associated with diet scores at a *P* < 1.08 × 10^−7^: 11 CpGs were associated with DASH, five CpGs with HPDI, and four CpGs with MMDs (*Table 2*; Supplementary material online, *Figure S5*). Two of those CpGs were common to the DASH diet and either MMDS or HPDI: cg03819286 (annotated to *MGRN1*) and cg13518625 (intergenic). Supplementary material online, *Table S1* shows 146 distinct diet-associated CpGs at a more lenient threshold (FDR < 5%). It should be noted that coefficients in the REGICOR-EPIC cohort were often of lower magnitude and precision (being the smallest sample included in this meta-EWAS), and that for cg03084350 and cg13518625, the heterogeneity was substantial (over 50%). Furthermore, EWAS coefficients were highly correlated for the three diet scores (see Supplementary material online, *Figure S6*), reflecting consistent associations between diet quality and methylation, regardless of the definition of a ‘healthy diet’ and the computation of the score. When restricting the meta-analysis to cohorts of European descent only (i.e. excluding estimates from the WHI, Supplementary material online, *Table S1*), all estimates were in the same direction as in the main analysis, and <10% showed an important attenuation of the coefficients (>25% attenuation). False discovery rate significance was lost in most cases due to the loss of power. Of the Bonferroni-significant CpGs, only cg02650017 (*PHOSPHO1*) was less strongly associated with diet scores, while the others displayed similar associations as in the main meta-analysis.

**Table 2:** Meta-epigenome-wide association study of diet quality in four cohorts of adults: top hit diet-associated CpGs (*P* < 1.08 × 10^−7^) and previously reported associations with cardiometabolic traits and other relevant

Diet score	CpG	CHR	Position (BP)	Gene	*β* ^a^	SE	*P*-value	*I* ^2b^	Previously reported association^c^					
									Inflammation	Adiposity	Lipids	CVD	Smoking	Other
DASH	cg05399785	1	3564031	*WDR8*	−0.059	0.011	4.62E−08	0	C-reactive protein (+)^48^	BMI (+)^49^		T2D (+)^50^ IHD (+)^50^	Smoking (+)^51^	COPD (+)^50^
DASH	cg12728588	1	36025489	*NCDN*	−0.057	0.011	8.71E−08	0	C-reactive protein (+)^48^					
DASH	cg03084350	3	38065265	*PLCD1*	−0.053	0.009	1.60E−09	0.557^d^						
DASH	cg02107842	5	66255772	*MAST4*	−0.044	0.008	1.59E−08	0						
MMDS	cg05575921	5	373378	*AHRR*	0.069	0.011	3.70E−10	0.089	C-reactive protein (−)^48^			CIMT (−)^52^ MI (−)^24^	Smoking (−)^51^	Lung dysfunction (+)^53^
HPDI	cg27395200	6	32942710	*BRD2*	−0.054	0.010	1.14E−08	0.003				Fasting insulin (+)^49^	Smoking (−)^51,54^	AD (+)^55^ COPD (−)^50^
DASH	cg08774868	7	80399952	*SEMA3C*	−0.063	0.011	2.92E−09	0.458					Smoking (+)^51^	
DASH	cg13518625	8	29522838	—	0.066	0.011	1.75E−09	0.510d		BMI (−)^51^		T2D (−)^50^ IHD (−)^50^	Smoking (−)^51^	COPD (−)^50^
MMDS					0.066	0.012	3.13E−08	0.488						
HPDI	cg23900905	8	82641291	—	0.077	0.014	1.37E−08	0						
DASH	cg23761815	10	73083123	*SLC29A3*	−0.054	0.009	1.03E−09	0.265	C-reactive protein (+)^48^			T2D (+)^50^		COPD (+)^50^
DASH	cg00574958	11	68607622	*CPT1A*	0.068	0.011	1.24E−09	0.022		BMI (−)^56^ WC (−)^12^	TG (−)^57^	T2D (−)^58^		
HPDI	cg02079413	11	2986505	*SNORA54*	−0.065	0.012	5.03E−08	0.478		BMI (+)^56^				
DASH	cg01678580	16	4674018	*MGRN1*	−0.052	0.009	3.61E−08	0		WC (+)^59^				
DASH	cg03819286	16	4673974	*MGRN1*	−0.047	0.008	2.20E−09	0.309						
HPDI					−0.052	0.008	5.00E−10	0						
HPDI	cg02650017	17	47301614	*PHOSPHO1*	0.068	0.012	1.75E−08	0.141	C-reactive protein (−)^48^	BMI (−)^12,56^	HDL-c (+)^57^	T2D (−)^58^		Crohn’s *d* (−)^60^
MMDS	cg18181703	17	76354621	*SOCS3*	0.078	0.012	4.08E−11	0.426	C-reactive protein (−)^48^	BMI (−)^61^		T2D (−)^58^	Smoking (−)^62^	Lung dysfunction (+)^53^
DASH	cg20761853	17	76850198	*TIMP2*	−0.056	0.010	6.81E−09	0.072						
MMDS	cg00711496	19	50191497	*PRMT1*	0.071	0.013	8.28E−08	0				SBP (−)^63^	Smoking (−)^51^	Crohn’s *d* (+)^64^

AD, Alzheimer’s disease; CpG, cytosine–phosphate–guanine; DASH, Dietary Approaches to Stop Hypertension; MMDS, Modified Mediterranean Diet Score; HPDI, Healthful Plant-based Diet Index; CHR, chromosome; BP, base pairs; CIMT, carotid intima–media thickness; BMI, body mass index; WC, waist circumference; HDL-c, HDL cholesterol; IHD, ischaemic heart disease; MI, myocardial infarction; SBP, systolic blood pressure; TG, triglycerides; T2D, type 2 diabetes.

^a^Regression coefficients are DNA methylation (*M*-value SD) change per 1 SD increase in diet scores from fixed effects meta-analysis (except when *I*^2^ > 0.50, where we report random effect meta-analysis estimate, indicated by the letter d) using Model 1 (adjusted for age, sex, blood cells, smoking, and technical variables).

^b^ *I* ^2^ for heterogeneity of coefficients across cohorts: ranges from 0 (absence of heterogeneity) to 1 (maximum heterogeneity).

^c^Direction of the previously reported associations of the CpG with cardiometabolic outcomes, smoking, and other outcomes (source: http://www.ewascatalog.org/).

When further adjusting for BMI (Model 2), we found that 12 CpGs displayed weaker coefficients of the association between diet quality and methylation, but that six CpGs (cg05575921, cg23900905, cg13518625, cg02650017, cg18181703, and cg00711496) showed only a slight attenuation of the association with MMDS or HPDI (*Table 3*). These CpGs were annotated to four different protein-coding genes and considered the ‘top’ diet-related CpGs.

**Table 3:** Consistent diet-associated CpGs after adjustment for body mass index (<10% attenuation)

Diet score	CpG	CHR	Position (BP)	Gene	*β* M1^a^	SE M1^a^	*P*-value M1^a^	*β* M2^b^	SE M2^b^	*P*-value M2^b^	*I* ^2^ M1^c^	*I* ^2^ M2^c^	% change in coefficient M1 to M2^d^
MMDS	cg05575921^e^	5	373378	*AHRR*	0.0688	0.0110	3.70E−10	0.0800	0.0212	1.66E−04	0.20	0	−16.2357
HPDI	cg23900905	8	82641291	—	0.0774	0.0136	1.37E−08	0.0771	0.0219	4.38E−04	0	0	0.3983
MMDS	cg13518625	8	29522838	—	0.0655	0.0118	2.62E−08	0.0685	0.0235	3.61E−03	0.46	0	−4.5291
HPDI	cg02650017	17	47301614	*PHOSPHO1*	0.0683	0.0121	1.75E−08	0.0646	0.0219	3.19E−03	0.14	0	5.3190
MMDS	cg18181703	17	76354621	*SOCS3*	0.0778	0.0116	2.09E−11	0.0824	0.0245	7.65E−04	0.29	0	−5.8260
MMDS	cg00711496	19	50191497	*PRMT1*	0.0710	0.0132	9.24E−08	0.0711	0.0248	4.11E−03	0	0	0.4841

M1, Model 1; M2, Model 2; CpG, cytosine–phosphate–guanine; DASH, Dietary Approaches to Stop Hypertension; MMDS, Modified Mediterranean Diet Score; HPDI, Healthful Plant-based Diet Index; CHR, chromosome.

^a^Regression coefficients are DNA methylation (*M*-value SD) change for per 1 SD increase in diet scores from fixed effects meta-analysis using Model 1 (non-adjusted for BMI).

^b^Regression coefficients are DNA methylation (*M*-value SD) change for per 1 SD increase in diet scores from fixed effects meta-analysis using Model 2 (adjusted for BMI).

^c^ *I* ^2^ for heterogeneity of coefficients across cohorts: ranges from 0 (absence of heterogeneity) to 1 (maximum heterogeneity).

^d^Change in the diet–CpG beta coefficient after adjustment for BMI (from Model 1 to Model 2). A positive value reveals attenuation, a null value reveals no change in coefficient, and a negative value reveals a coefficient of stronger magnitude after adjustment for BMI.

^e^Hypomethylation of cg05575921 has been consistently associated with smoking.

Because the MMDS-associated CpG cg05575921 mapping to the *AHRR* gene has been previously strongly associated with smoking, we conducted stratified analysis by smoking status. We found that the association was null in non-smokers and only apparent in ever smokers (see Supplementary material online, *Figure S7*).

Among the 18 CpGs, 16 were annotated to 15 protein-coding genes. Some of these genes are involved in metabolic pathways including fatty acid beta-oxidation and calcification (*CPT1A* and *PHOSPHO1*) and in some cancers (*PRMT1*). *SOCS3* has also been reported to be a major regulator in inflammation and infection. According to the GTEx expression data set, these annotated genes are differentially expressed in different tissues, with a majority of genes expressed in spleen, lung, and whole blood (see Supplementary material online, *Figure S8*).

### Association between diet-associated CpGs and cardiometabolic markers

Out of the 18 Bonferroni-significant CpGs, methylation levels corresponding to higher diet quality were associated with lower C-reactive protein levels (six CpGs), lower BMI (six CpGs), lower waist circumference (two CpGs), lower risk of T2D (three CpGs), lower triglycerides (one CpG), higher HDL cholesterol (one CpG), lower SBP (one CpG), lower carotid intima–media thickness (one CpG), lower risk of myocardial infarction (one CpG), better lung function (two CpGs), and lower risk of Crohn’s disease (two CpGs). Methylation levels linked to better diet quality were also inversely related to odds of smoking in seven CpGs (*Table 2*).

### Enrichment analysis of diet-associated CpGs

We only found significant enrichment for the genes mapped to the FDR-significant diet-associated CpGs using the ConsensusPathDB (*Table 4*) but not with the annotations from GO, KEGG, or MSigDB (see Supplementary material online, *Tables S2–S4*). The terms from protein complex–based sets with greater overrepresentation of the diet-related genes were the SKI–SMAD3 complex, a family of signal transducer and transcription modulator that interfaces multiple signalling pathways, involved in regulating cell proliferation, apoptosis, and differentiation.^65^ Besides, we found enrichment for R-spondin (RSPO) proteins and NFKB1–NFKB2–RELA–RELB complex. We also obtained significant results in the enrichment of GO Level 2 for several biological processes (see Supplementary material online, *Table S5*). Although not reaching FDR statistical significance, the most represented pathways across databases (GO, KEGG, and manually curated pathways) were signalling of TGF-beta receptor, MAPK, interleukin-1, interleukin-6, VEGF-A/VEGFR2, SMAD2/3:SMAD4 transcriptional activity, and fatty acid metabolism (see Supplementary material online, *Tables S2*, *S3*, and *S6*). When we analysed gene position (see Supplementary material online, *Figure S9A* and *Table S7*), we found TSS1500 and gene body region being significantly overrepresented, whereas TSS200 was underrepresented. Regarding CpG island relative positions (see Supplementary material online, *Figure S9B* and *Table S8*), N shelf, S-shore and body it flanks, and Open Sea were significantly overrepresented, whereas island was underrepresented. As for the chromatin state analysis, transcription-related (Tssa Flnk, Tx, Tx Flnk, and TxWk) and enhancers (Enh and EnhG) were overrepresented (see Supplementary material online, *Figure S9C* and *Table S9*).

**Table 4:** Gene enrichment analysis in protein complex–based sets (ConsensusPathDB) of the false discovery rate–significant diet-associated CpGs

Protein complex^a^	All entities (*n*)^b^	Overlapping entities (*n*)^c^	*P*-value^d^	FDR^e^
SKI–SMAD3 complex	2	2	4.E−05	4.E−04
SKI–SMAD3–SMAD4 complex	3	2	1.E−04	4.E−04
SMAD3–SKI–NCOR complex	3	2	1.E−04	4.E−04
SMAD3–cSKI–SIN3A–HDAC1 complex	4	2	2.E−04	6.E−04
LGR4–RSPO supercomplex	11	2	2.E−03	4.E−03
CHUK–NFKB2–REL–IKBKG–SPAG9–NFKB1–NFKBIE–COPB2–TNIP1–NFKBIA–RELA–TNIP2 complex	12	2	2.E−03	4.E−03
R-smad:smad4 complex:corepressor:deacetylase:responsive element	21	2	0.01	0.01
Cytokine receptor:SOCS3	40	2	0.03	0.03

^a^FsetID and URLs to protein complex–based set term visualization can be found in Supplementary material online, *Table S10*.

^b^Number of genes in the protein complex term.

^c^Number of genes annotated to the list of the FDR-significant diet-related CpGs.

^d^ *P*-value for overrepresentation of the protein complex term within the CpG list.

^e^False discovery rate (adjusted *P*-value).

### Mendelian randomization analyses

There were genetic instruments available in the GoDMC database only for 10 out of the 18 Bonferroni-significant CpGs (four out of the ‘top’ six CpGs). Mendelian randomization suggested that genetically determined methylation levels corresponding to lower diet quality at cg02079413 (*SNORA54*), cg02107842 (*MAST4*), and cg23761815 (*SLC29A3*) were causally associated with higher BMI and at cg05399785 (*WDR8*) with greater SBP (*Table 5*). Mendelian randomization also showed methylation levels associated with higher diet quality at cg00711496 (*PRMT1*) to be causally associated with lower BMI, T2D risk, and CHD risk and at cg0557921 (AHRR) with lower CHD risk [although weakly, risk ratio (RR) = 0.98 (0.96; 1.00), *P* = 0.05]. Nonetheless, we also observed results that contradict our hypothesis and the observational evidence with disease risk: methylation levels reflecting higher diet quality at cg02650017 (*PHOSPHO1*) were associated with higher T2D risk, and methylation levels reflecting lower diet quality at cg27395200 (*BRD2*) were associated with lower CHD risk and at cg23761815 (*SLC29A3*) were associated with lower SBP and lower T2D risk. For other traits and other CpGs, results were either non-significant or did not meet all MR assumptions.

**Table 5:** Mendelian randomization analyses between diet-related CpGs and four cardiometabolic traits and coronary heart disease

CpG and outcome	SNPs	Assoc. MR (IVW)^a^	Validated MR assumptions^b^			*F*-stat	MR estimate: IVW	
			MR-Egger intercept	MR estimates	SNP heterog.		Beta/OR (95% CI)	*P*-value
**cg00711496 (PRMT1) association with diet +**								
Body mass index	14	**—**	Yes	Yes	Yes	582	−0.026 (−0.032; −0.020)	<1.0E–5
Systolic blood pressure	15		Yes	No	No	558	−0.13 (−0.23; −0.032)	0.009
Triglycerides	14					558	−6 × 10^−4^ (−0.004; 0.003)	0.710
Type 2 diabetes^c^	15	**—**	Yes	Yes	Yes	558	0.98 (0.97; 0.98)	<1.0E−5
Coronary heart disease^c^	14	**—**	Yes	Yes	Yes	558	0.98 (0.96; 0.99)	0.01
**cg02079413 (SNORA54) association with diet −**								
Body mass index	33	**+**	Yes	Yes	Yes	869	0.012 (0.010; 0.014)	<1.0E−5
Systolic blood pressure	37					935	0.018 (−0.026; 0.062)	0.431
Triglycerides	37		Yes	Yes	No	935	−0.003 (−0.005; −6 × 10^−4^)	0.014
Type 2 diabetes^c^	37		Yes	Yes	No	935	0.98 (0.97; 0.99)	<1.0E−5
Coronary heart disease^c^	32					935	1.00 (1.00; 1.01)	0.203
**cg02107842 (MAST4) association with diet −**								
Body mass index	5	**+**	Yes	Yes	Yes	359	0.011 (0.005; 0.017)	<0.001
Systolic blood pressure	6					327	−0.009 (−0.21; 0.20)	0.929
Triglycerides	6		No	No	No	327	−0.015 (−0.026; −0.003)	0.012
Type 2 diabetes^c^	6		Yes	No	Yes	327	0.99 (0.98; 1.00)	0.004
Coronary heart disease^c^	5		Yes	No	Yes	327	0.96 (0.94; 0.99)	0.011
**cg02650017 (PHOSPHO1) association with diet +** ^d^								
Body mass index	17		No	No	No	569	−0.015 (−0.020; −0.009)	<1.0E−5
Systolic blood pressure	19					534	−0.002 (−0.16; 0.15)	0.981
Triglycerides	18		Yes	Yes	No	534	0.014 (0.007; 0.022)	<0.001
Type 2 diabetes^c^	19	**+**	Yes	Yes	Yes	534	1.03 (1.02; 1.05)	<1.0E−5
Coronary heart disease^c^	17		No	Yes	No	534	0.83 (0.81; 0.85)	<1.0E−5
**cg03084350 (PLCD1) association with diet −**								
Body mass index	10		Yes	No	Yes	149	0.010 (0.002; 0.019)	0.022
Systolic blood pressure	13					142	0.029 (−0.17; 0.22)	0.768
Triglycerides	13					142	0.003 (−0.005; 0.011)	0.486
Type 2 diabetes^c^	13					142	0.99 (0.94; 1.04)	0.641
Coronary heart disease^c^	10					142	1.00 (0.96; 1.03)	0.806
**cg05399785 (WDR8) association with diet −**								
Body mass index	15		Yes	Yes	No	421	0.007 (6 × 10^−4^; 0.013)	0.031
Systolic blood pressure	16	**+**	Yes	Yes	Yes	406	0.10 (0.009; 0.19)	0.031
Triglycerides	16		Yes	Yes	No	388	−0.009 (−0.014; −0.004)	<0.001
Type 2 diabetes^c^	16		Yes	No	Yes	388	0.99 (0.98; 1.00)	0.01
Coronary heart disease^c^	16					388	0.99 (0.97; 1.01)	0.389
**cg05575921 (AHRR) association with diet +** ^d^								
Body mass index	22					222	−0.007 (−0.014; 8 × 10^−4^)	0.08
Systolic blood pressure	22					219	0.20 (−0.005; 0.40)	0.055
Triglycerides	22					219	0.002 (−0.001; 0.006)	0.197
Type 2 diabetes^c^	22					219	0.99 (0.96; 1.02)	0.525
Coronary heart disease^c^	17	**—**	Yes	Yes	Yes	219	0.98 (0.96; 1.00)	0.052
**cg13518625 (intergenic) association with diet +** ^d^								
Body mass index	9		Yes	No	No	263	0.019 (0.009; 0.029)	<0.001
Systolic blood pressure	11					272	0.077 (−0.14; 0.30)	0.49
Triglycerides	11					272	−0.028 (−0.067; 0.011)	0.164
Type 2 diabetes^c^	11		Yes	No	Yes	272	1.09 (1.06; 1.12)	<1.0E−5
Coronary heart disease^c^	8		Yes	No	Yes	272	1.08 (1.05; 1.11)	<1.0E−5
**cg23761815 (SLC29A3) association with diet −**								
Body mass index	15	**+**	Yes	Yes	Yes	111	0.023 (0.015; 0.030)	<1.0E−5
Systolic blood pressure	15	**—**	Yes	Yes	Yes	111	−0.27 (−0.40; −0.14)	<0.001
Triglycerides	15					111	0.002 (−0.004; 0.008)	0.584
Type 2 diabetes^c^	15	**—**	Yes	Yes	Yes	111	0.92 (0.90; 0.94)	<1.0E−5
Coronary heart disease^c^	12					111	1.05 (0.98; 1.12)	0.134
**cg27395200 (BRD2) association with diet −**								
Body mass index	2		?	?	?	226	0.038 (0.028; 0.049)	<1.0E−5
Systolic blood pressure	7					189	−0.21 (−0.61; 0.19)	0.293
Triglycerides	7					189	−0.002 (−0.020; 0.015)	0.806
Type 2 diabetes^c^	7					189	0.99 (0.96; 1.02)	0.511
Coronary heart disease^c^	6	**—**	Yes	Yes	Yes	189	0.97 (0.94; 0.99)	0.019

MR, Mendelian randomization; IVW, inverse-variance weighted; SNP, single nucleotide polymorphism; OR, odds ratio; CI, confidence interval; *F*-stat, *F*-statistic.

^a^MR assumptions #1 [the genetic instruments used are robustly related to the exposure (*F*-stat > 10)] and #2 (no confounding of the genetic instrument–outcome associations is present) were verified for all CpGs. Assumption #3 (the genetic instruments are exclusively linked to the outcome by the exposure of interest) is verified if there is no evidence of pleiotropy. Pleiotropy can be detected if (i) the MR-Egger method shows a non-zero intercept; (ii) there is lack of concordance among MR estimates (magnitude, direction) in inverse-variance weighted and the alternative methods; and (iii) between-SNP heterogeneity is observed according to Cochran’s *Q* and Rücker’s *Q*. We provide here a summary of whether each of these three assumptions on absence of pleiotropy are met (yes) or not (no). The information to reach these conclusions can be found in Supplementary material online, *Table S11*.

^b^Even if the IVW estimate is significant, we do not consider this result valid if the one or more of the MR assumptions are not verified.

^c^For type 2 diabetes and coronary heart disease, the estimate is an OR/RR.

^d^‘Top’ diet-related CpG for which the association with diet was not attenuated after adjustment for BMI.

## Discussion

In this meta-EWAS, we found 18 CpG sites showing differential methylation levels associated with three diet quality scores. Mendelian randomization analyses suggest that some of these CpGs may be causally associated with BMI, blood pressure, and T2D, implying that DNAm may be a mechanism by which diet quality influences cardiometabolic health.

Some of our findings replicated previous diet quality EWAS results. Three CpGs were previously associated with diet quality scores in the same direction as in our study: cg03084350 (*PLCD1*),^10^ cg02079413 (*SNORA54*), and cg18181703 (*SOCS3*).^9^ Moreover, the positive association between methylation at cg00574958 (*CPT1A*) and a greater DASH score (a typically low-fat diet) was consistent with previous findings of a positive association with dietary carbohydrates and negative with dietary fat intake.^66^ Mediation analysis in the same study revealed significant indirect effects through the mediator cg00574958, meaning that fat and carbohydrate intake influences the risk of metabolic diseases (BMI, diabetes, triglycerides, SBP, and metabolic syndrome) through CPT1A-cg00574958 methylation. However, no genetic instruments were available for this CpG to perform an MR analysis. Our results are observational, and only one study to date has investigated the effect of a Mediterranean diet intervention in a subsample (*n* = 36) of the PREDIMED trial^67^: we did not replicate the findings of that study which showed the intervention was associated with change in methylation at cg01081346 and cg17071192.

The majority of the associations between diet scores and DNAm were attenuated when BMI was included as a confounder, which suggests that BMI explains an important part of the observed associations and that the observed differential methylation may be a consequence of adiposity. However, the associations between diet scores and six CpGs displayed coefficients of similar magnitude (or even stronger) regardless of BMI, indicating that diet was associated with methylation at those CpGs independently of BMI, making them robust methylation signatures of diet quality.

One CpG, cg05575921, maps to *AHHR*, and hypermethylation at this CpG has been shown to be associated with lower inflammation, carotid intima–media thickness, and higher risk of myocardial infarction,^68^ while hypomethylation has been consistently associated with smoking.^69^ Therefore, it is possible that hypermethylation might be more driven by non-smoking status than by diet quality, as it was also suspected in the recent EWAS of coffee consumption,^8^ although smoking status was adjusted for. However, when stratifying by smoking status, the association with diet was only apparent in smokers: this might suggest that diet-related hypermethylation only occurs at this site if already hypomethylated (as typically observed for smokers), as a potential protective mechanism.^70^ Our result aligns with those of a recent Japanese study, whereby fruit intake and coloured vegetable intake were associated with hypermethylation at this CpG only in current and ex-smokers.^71^

Among other CpGs of interest, cg18181703 is annotated to *SOCS3*, a gene involved in immune system regulation. This CpG is positively associated with diet quality, and inverse associations between methylation at this CpG and C-reactive protein,^48^ BMI,^56,61,72^ and T2D risk^58^ have been reported. This CpG was previously identified as associated with diet quality, and all-cause mortality,^9^ suggesting this epigenetic modification by diet might involve inflammation. Unfortunately, we could not perform MR due to the lack of mQTLs for this CpG. Similarly, methylation at CpGs such as cg07573872, cg12269535, and cg26470501 has been associated with lower BMI and C-reactive protein levels in previous studies.^48,49,56^ Moreover, cg07573872 maps to *SBNO2*, cg12269535 maps to *SRF*, and cg26470501 maps to *BCL3*, which are all genes implicated in inflammation response.^54,73^

Functional analyses suggested an overrepresentation of diet-related genes in the SKI–SMAD3 complex and TGF-β signalling pathway. The SMAD complex interfaces signalling pathways targeting gene promoters and is required for the TGF-β transcriptional responses.^74^ Both SMADs and TGF-β proteins, as well as the transcription factor NF-κB, MAPK, and IL-1, which were also enriched, are involved in inflammatory signalling and cardiovascular diseases.^75–77^ Another interesting enriched complex was RSPO, which can agonize the Wnt/β-catenin signalling pathway which controls vascular smooth muscle cell phenotypic modulation in cardiometabolic disease.^78^ Finally, there was an overrepresentation of genes involved in the vascular endothelial growth factor VEGF-A/VEGFR2 pathways, which are involved in atherosclerosis.^79^

Our findings suggest that changes in DNAm may mediate the protective effects of diet on chronic disease. Methylation levels corresponding to higher diet quality were associated in previous studies with lower levels of risk factors for cardiometabolic disease (adiposity, low-grade inflammation, dyslipidaemia, T2D, and ischaemic heart disease), better lung function, and lower risk of other chronic inflammatory diseases (e.g. Crohn’s disease). Furthermore, MR analysis supported causal effects of five CpGs [cg02079413 (*SNORA54*), cg02107842 (MAST4), cg05399785 (*WDR8*), cg23761815 (*SLC29A3*), and cg00711496 (*PRMT1*)] on BMI, of cg00711496 (*PRMT1*) on T2D, of two CpGs [cg00711496 (*PRMT1*) and cg05575921 (AHRR)] on CHD, and of cg05399785 (*WDR8*) on SBP. *PRMT1* expression is elevated in certain conditions, such as cancer, and in several tissues, such as blood vessels, and it plays an important role in immunity and cardiovascular disease.^80^ Therefore, this diet-modifiable CpG might be an interesting target as it lies on the pathway between diet quality and various cardiometabolic traits. Nonetheless, MR evidence also supports causal associations between three CpGs and cardiometabolic traits in which better diet quality DNAm levels were associated with worse cardiometabolic health markers, which were opposite to what was found in observational EWAS, in particular for cg23761815 (SLC29A3) and cg02650017 (*PHOSPHO1*) in relation to T2D risk. However, it should be noted that when restricting the analysis to cohorts of European ancestry, cg02650017 was less strongly associated with diet, which might indicate an influence of ethnicity in this association, and the use of European summary statistics in the MR might not be appropriate. Moreover, methylation at cg27395200 (BRD2) was negatively and consistently associated with healthy diet in the present meta-EWAS but with various unhealthy behaviours (smoking^51^ and alcohol consumption^54^) previously. Higher methylation at this CpG was associated with lower CHD risk in MR analyses, whereas it was associated with higher levels of fasting insulin^81^ and higher risk of Alzheimer’s disease^55^ but lower risk of COPD^50^ in previous EWASs. Therefore, the methylation at this CpG in response to the environment and its role in disease aetiology is likely complex and involves different metabolic pathways.

Our study has various strengths, including the large sample size (*n* = 5274) from four well-characterized cohorts. While the FHS and WHI samples were used in previous diet quality EWASs,^9,10^ their results were not meta-analysed and focused on MMDS and AHEI, so the examination of the DASH and HPDI scores is unique to the present study. Moreover, unlike the largest EWAS on diet published to date,^9^ we included smoking as a confounder variable due to its effect on DNAm, making our results less biased. Another strength is the use of both cross-sectional (WHI and Airwave) and longitudinal (FOS and REGICOR) designs: the top hit associations were consistent in direction and magnitude as seen in the forest plots, which indicates robustness of these findings and partly rules out reverse causality bias. Similarly, WHI is ethnically diverse and comprise only women, and the AIRWAVE study is a younger population, but these differences did not result in distinctive patterns of diet–CpG associations for these two populations, indicating robustness of our findings. However, our study also has limitations. First, dietary exposures were not calculated using the same sources of information (AIRWAVE used 7-day food records while the other three cohorts used less precise food frequency questionnaires) and the same food items, so they might not reflect diet quality the same way. We tried to overcome this limitation by using widely used, replicable, validated, food-based dietary scores that do not include components on micronutrient intakes (except for sodium in the DASH score), which are notoriously unreliably estimated with food frequency questionnaires.^82^ Second, DNAm was measured in blood, whereas the biological impact of diet may be more relevant to measure in specific tissues such as adipose tissue, gastrointestinal, or liver cells.^83^ Third, our participants were middle aged adults of mostly European ancestry, which would limit the generalizability of our findings and might include selection bias. Studies of greater sample size with more diverse ethnicity representation are therefore necessary. Fourth, although MR comes as a great advance in observational epidemiology to investigate the causal effects of a wide range of exposures, there are some limitations associated to the fact that it assumes a relationship between the exposure, outcome, and instrument variable (genetic variants) to be true. Nonetheless, we have thoroughly tested MR assumptions by conducting different sensitivity analyses.

To conclude, this study found 18 diet-associated CpGs, five of which replicating previous findings with diet quality. Twelve of these CpGs showed associations with cardiometabolic traits in previous studies, particularly inflammation, adiposity, and T2D, and our MR analysis revealed some potential causal link with adiposity, T2D, blood pressure, and coronary artery disease. We demonstrate here that integrating information on diet quality and epigenetic markers has the potential to help understand the mechanisms that underlie the effect of diet on cardiometabolic health.

## Supplementary Material

zwad317_Supplementary_Data

## Data Availability

The R code for the analysis is available in the following Github repository: https://github.com/jorgedb98/B64_DIAMETR.git. Both FOS and WHI data are available in the database of Genotypes and Phenotypes (http://dbgap.ncbi.nlm.nih.gov; Project #9047). AIRWAVE data are available after approval by Dementia Platform UK (Study #0438) on https://portal.dementiasplatform.uk/. REGICOR data are available from authors upon reasonable request.
