# Supplementary material for: Blood DNA methylation signature of diet quality and association with cardiometabolic traits
Source: Eur J Prev Cardiol. 2023 Oct 4;31(2):191–202. doi: 10.1093/eurjpc/zwad317 (PMC10809172; doi:10.1093/eurjpc/zwad317)

Figure S1: Venn diagram of the number of CpGs available in each sample

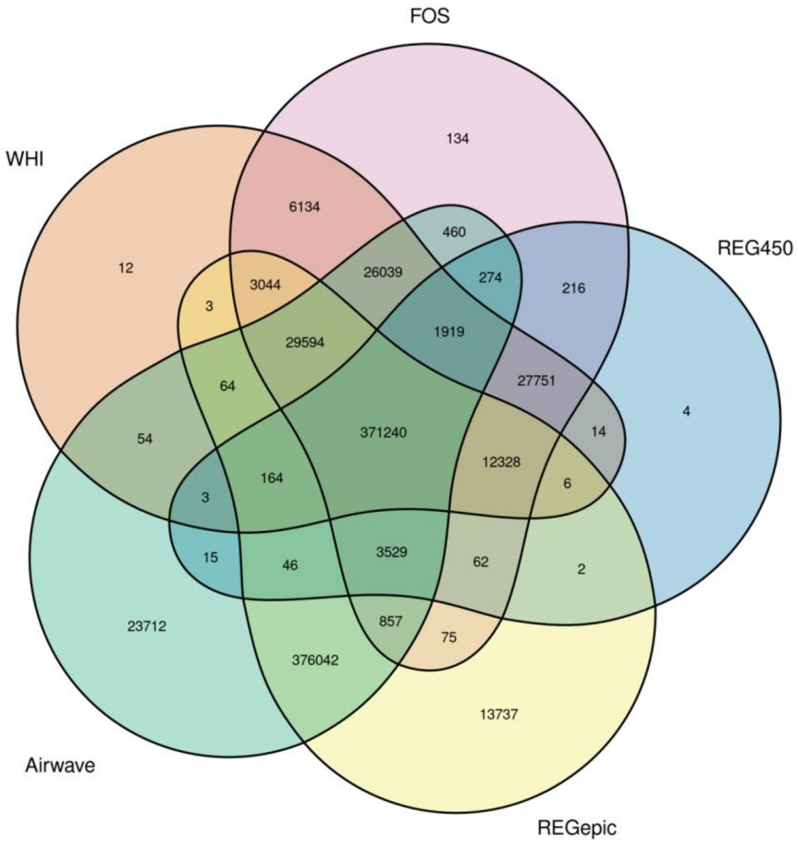

Figure S2: Q-Q plots with each diet score and in each cohort, before (left panel) and after (right panel) correction using *bacon.* (Pages 2-16)

AIRWAVE

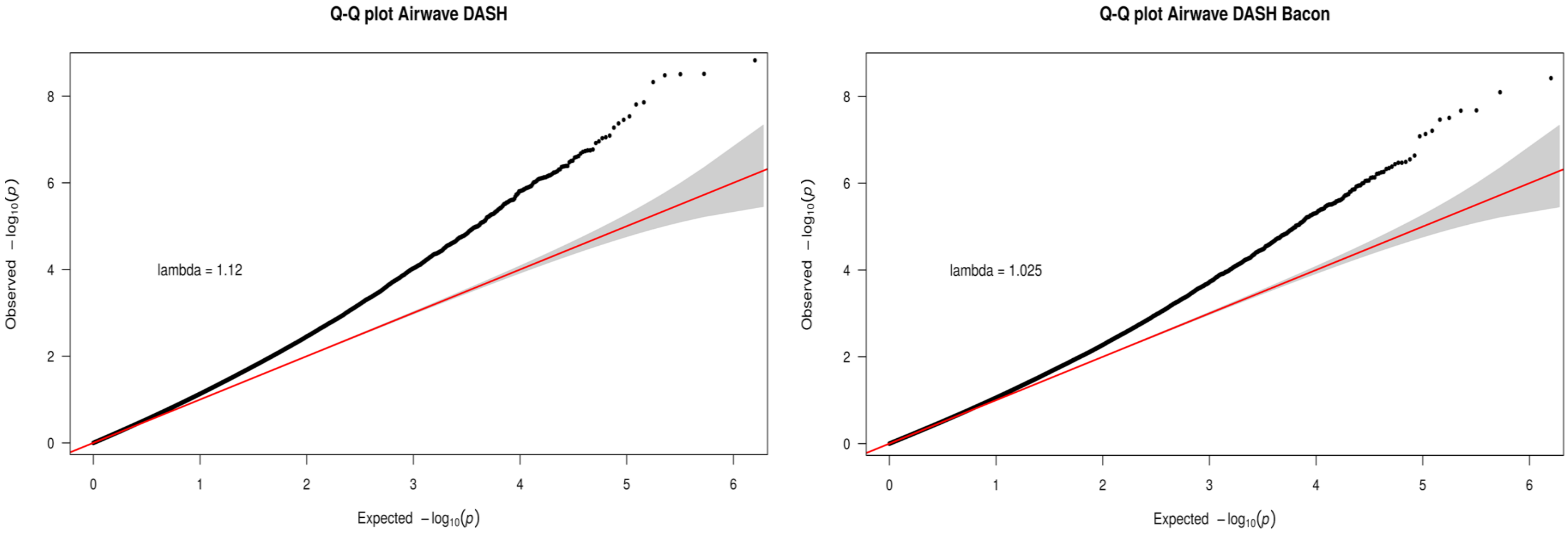

AIRWAVE

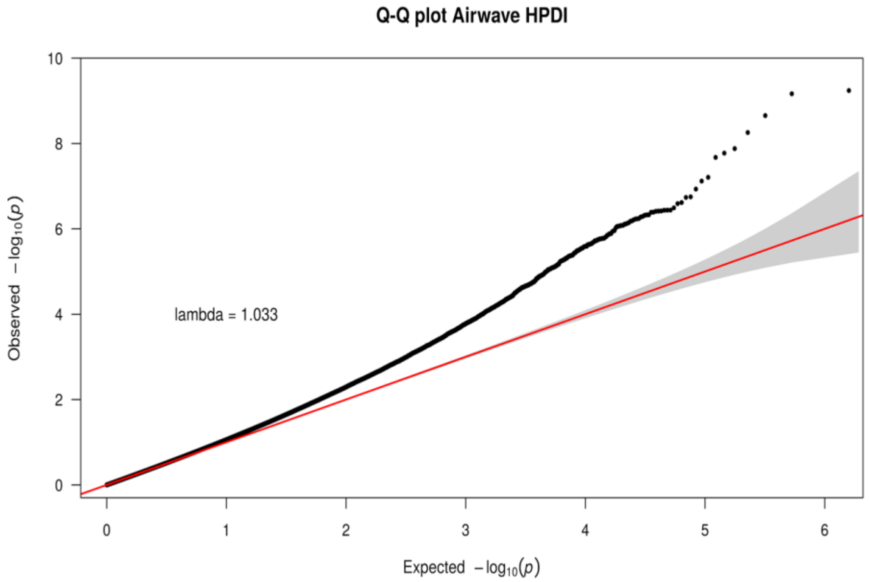
Q-Q plot Airwave HPDI
lambda = 1.033
Observed $-\log_{10}(p)$
Expected $-\log_{10}(p)$
0
2
4
6
8
10
0
1
2
3
4
5
6

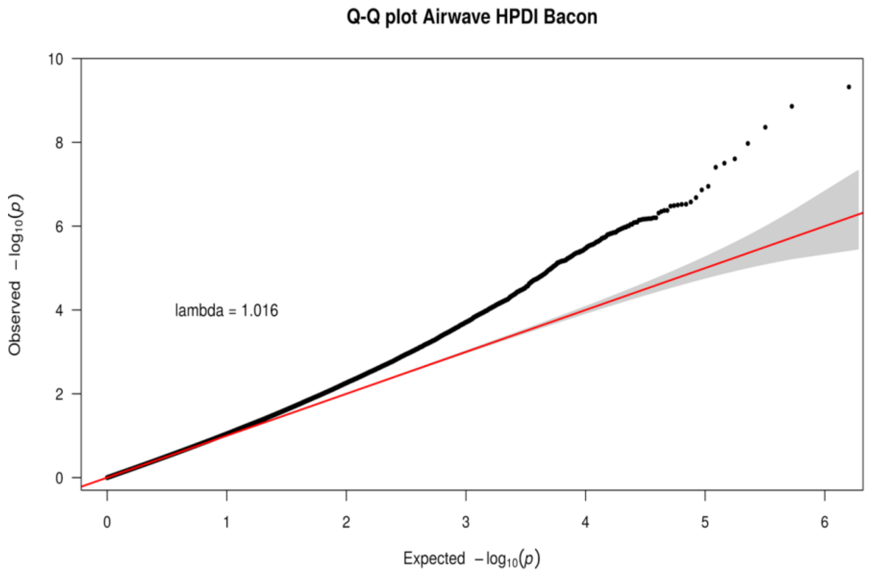
Q-Q plot Airwave HPDI Bacon
lambda = 1.016
Observed $-\log_{10}(p)$
Expected $-\log_{10}(p)$
0
2
4
6
8
10
0
1
2
3
4
5
6

AIRWAVE

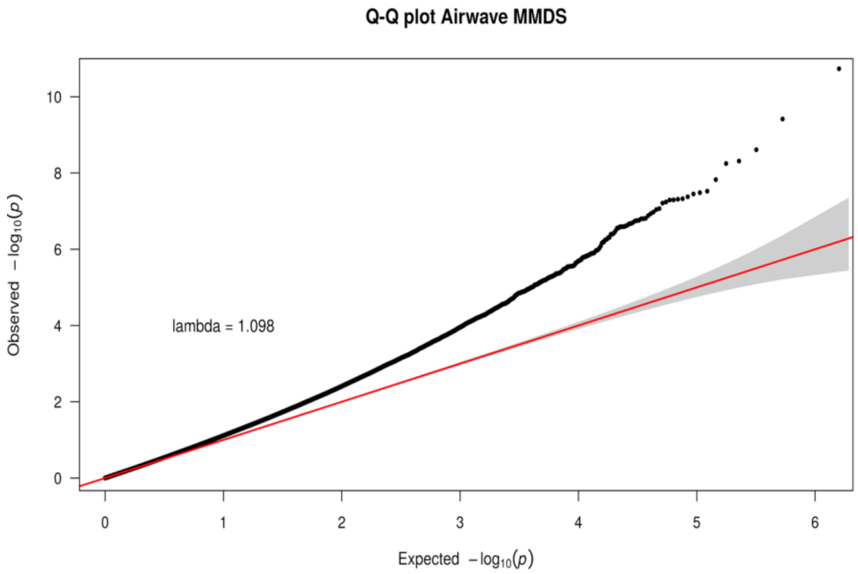
Q-Q plot Airwave MMDS
lambda = 1.098
Observed −log10(p)
Expected −log10(p)
0
2
4
6
8
10
0
1
2
3
4
5
6

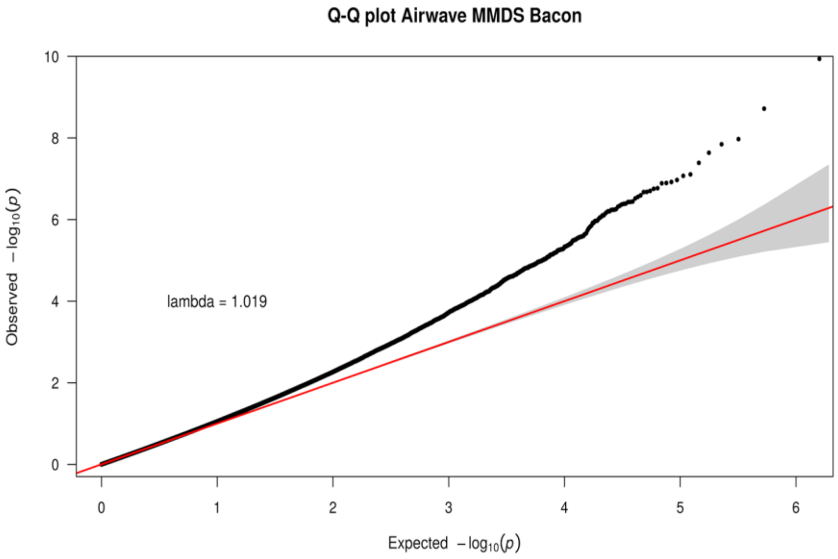
Q-Q plot Airwave MMDS Bacon
lambda = 1.019
Observed −log10(p)
Expected −log10(p)
0
2
4
6
8
10
0
1
2
3
4
5
6

FOS

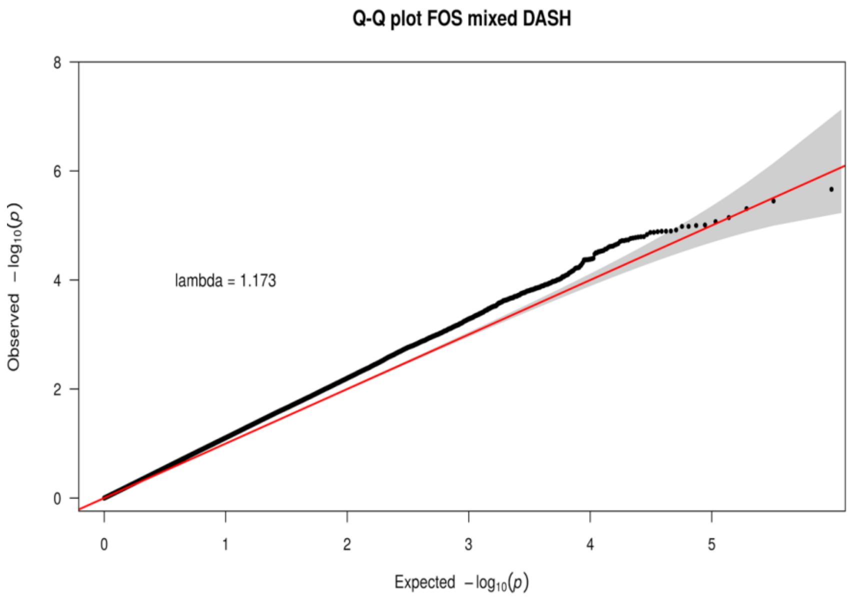
Q-Q plot FOS mixed DASH
lambda = 1.173
Observed $-\log_{10}(p)$
Expected $-\log_{10}(p)$
0
2
4
6
8
0
1
2
3
4
5

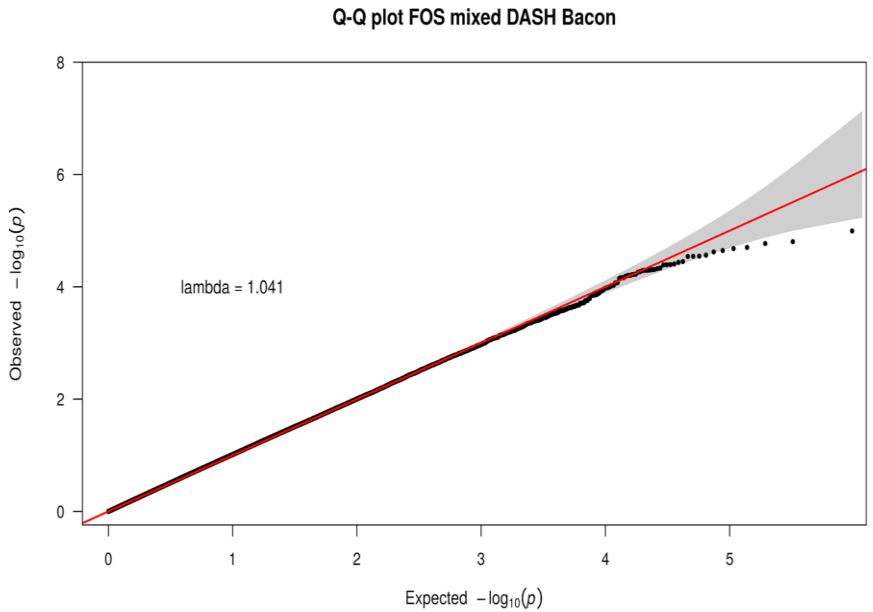
Q-Q plot FOS mixed DASH Bacon
lambda = 1.041
Observed $-\log_{10}(p)$
Expected $-\log_{10}(p)$
0
2
4
6
8
0
1
2
3
4
5

FOS

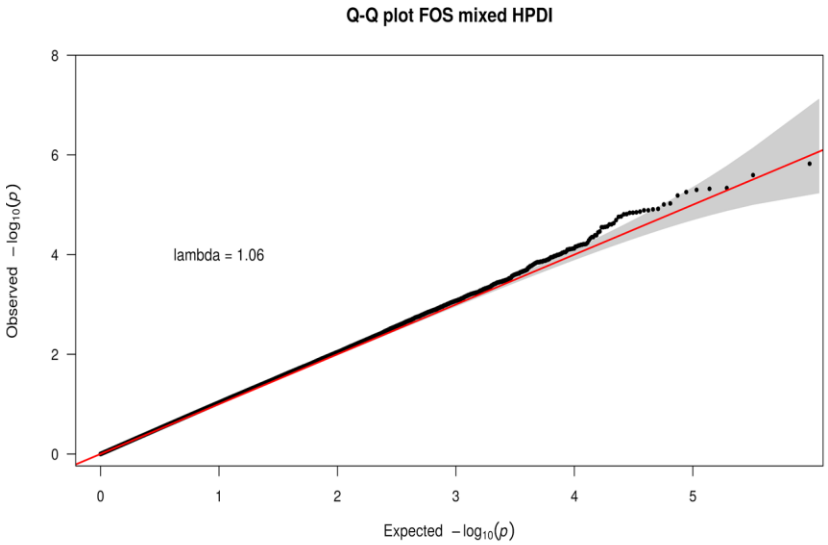
Q-Q plot FOS mixed HPDI
lambda = 1.06
Observed − log10(p)
Expected − log10(p)
0
2
4
6
8
0
1
2
3
4
5

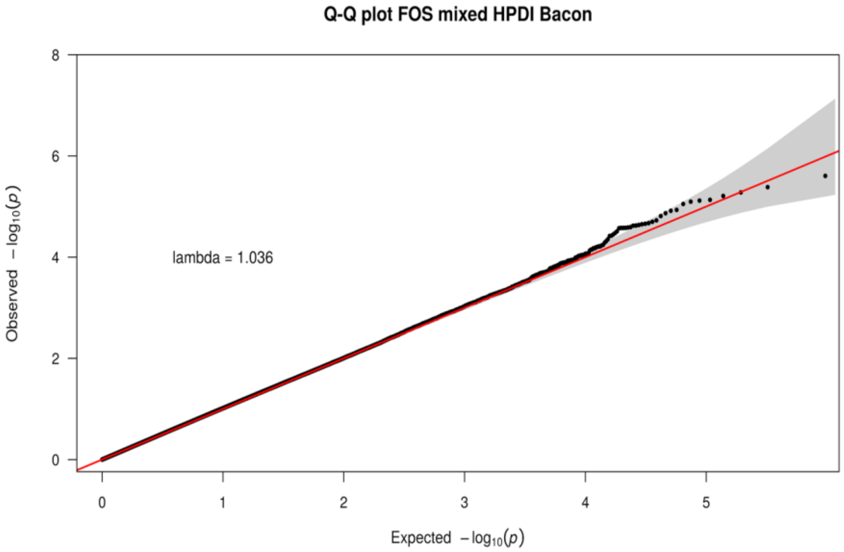
Q-Q plot FOS mixed HPDI Bacon
lambda = 1.036
Observed − log10(p)
Expected − log10(p)
0
2
4
6
8
0
1
2
3
4
5

FOS

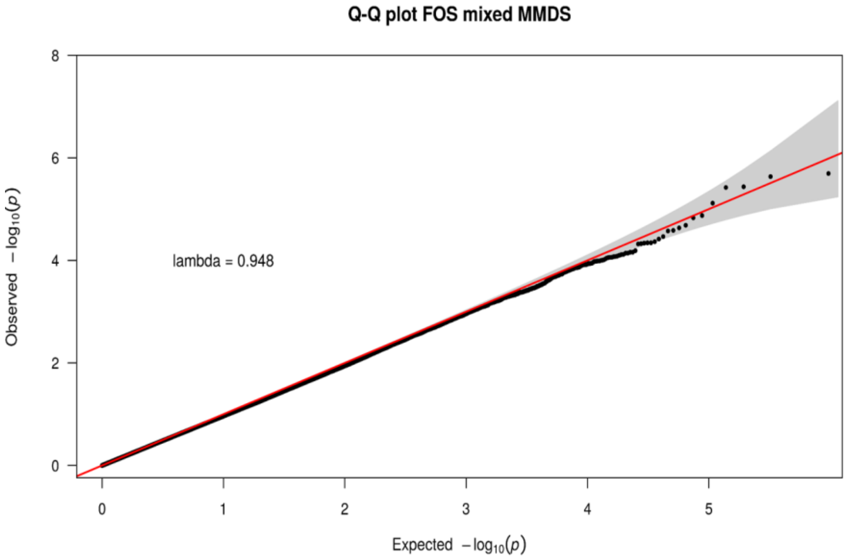

Q-Q plot FOS mixed MMDS
lambda = 0.948
Observed −log10(p)
Expected −log10(p)

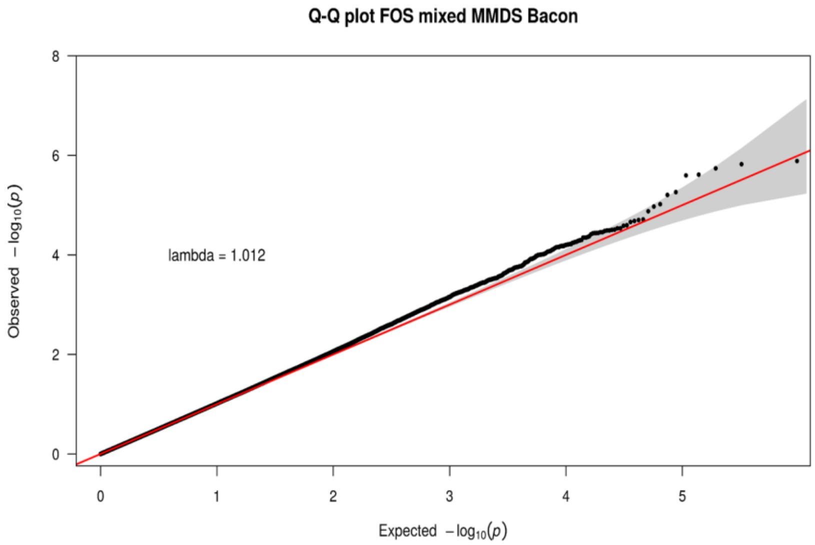

Q-Q plot FOS mixed MMDS Bacon
lambda = 1.012
Observed −log10(p)
Expected −log10(p)

WHI

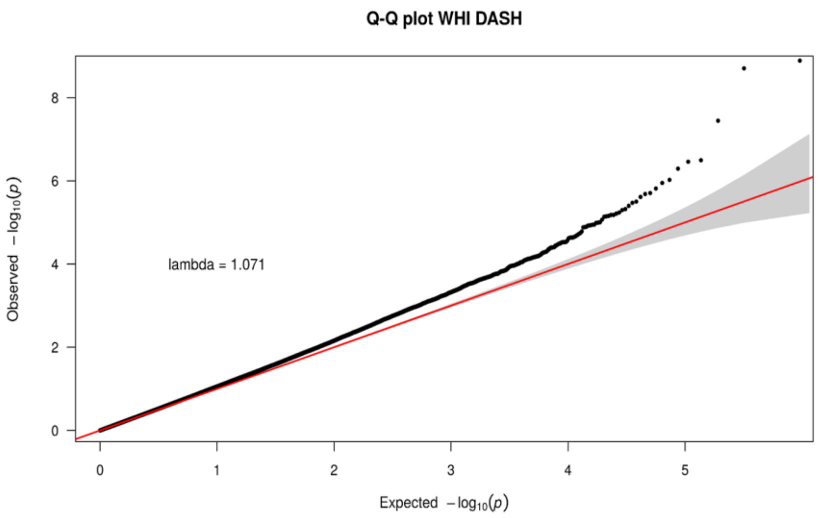
Q-Q plot WHI DASH
lambda = 1.071
Observed $-\log_{10}(p)$
Expected $-\log_{10}(p)$
0
2
4
6
8
0
1
2
3
4
5

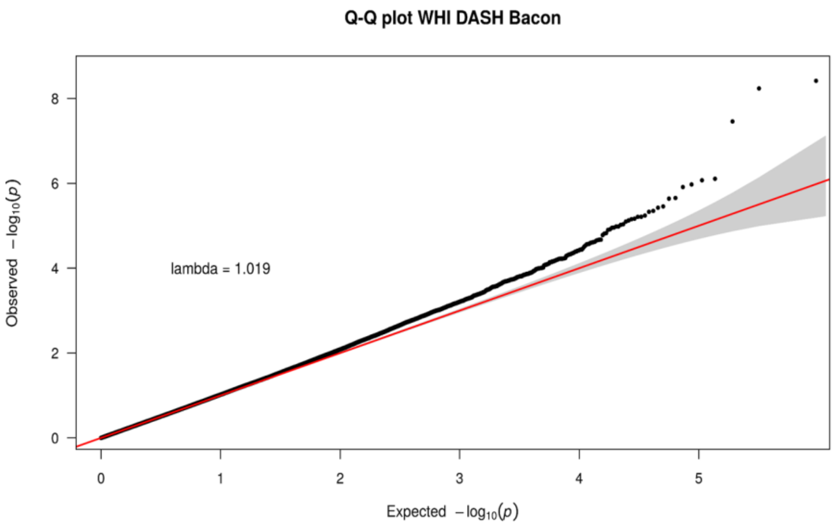
Q-Q plot WHI DASH Bacon
lambda = 1.019
Observed $-\log_{10}(p)$
Expected $-\log_{10}(p)$
0
2
4
6
8
0
1
2
3
4
5

WHI

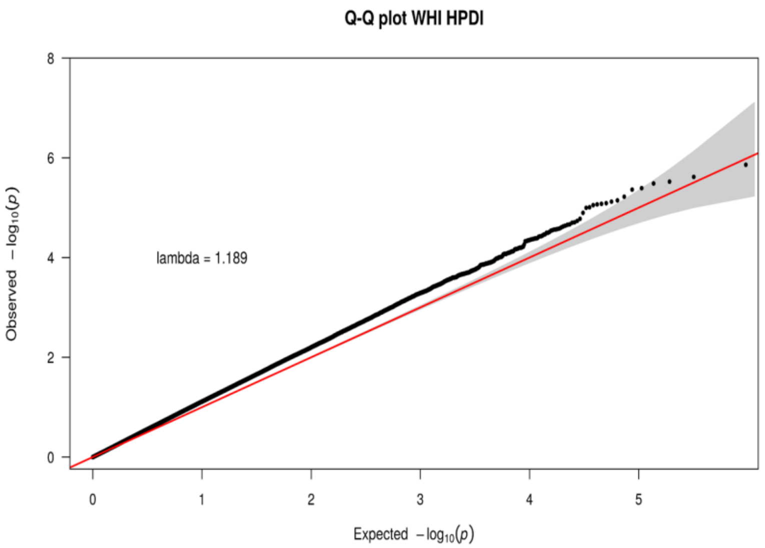
Q-Q plot WHI HPDI
lambda = 1.189
Observed − log10(p)
Expected − log10(p)
0
2
4
6
8
0
1
2
3
4
5

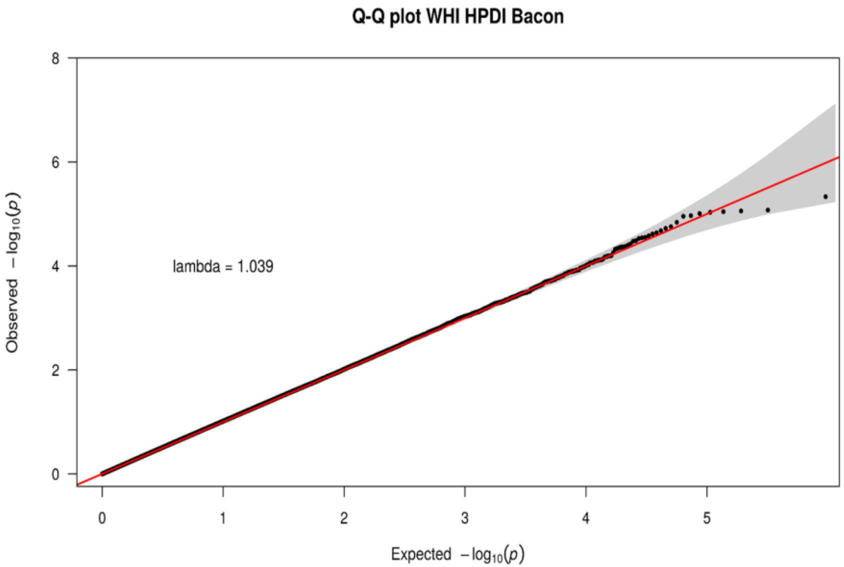
Q-Q plot WHI HPDI Bacon
lambda = 1.039
Observed − log10(p)
Expected − log10(p)
0
2
4
6
8
0
1
2
3
4
5

WHI

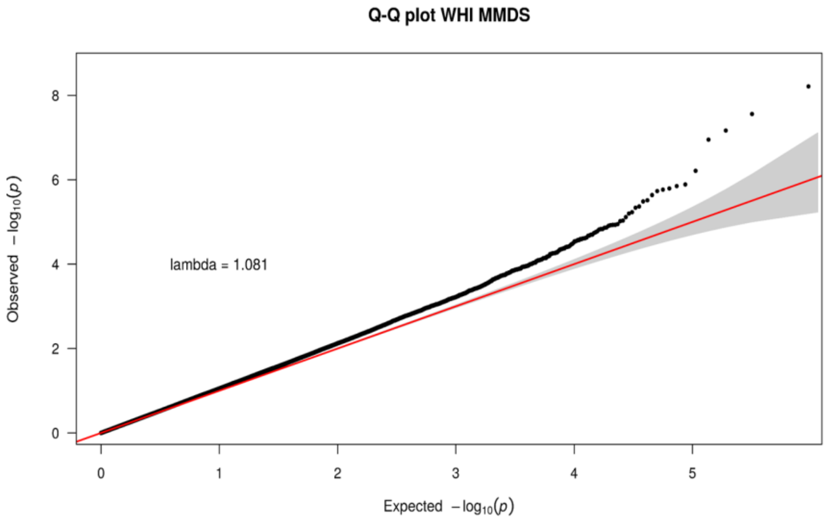
Q-Q plot WHI MMDS
lambda = 1.081
Observed $-\log_{10}(p)$
Expected $-\log_{10}(p)$
0
2
4
6
8
0
1
2
3
4
5

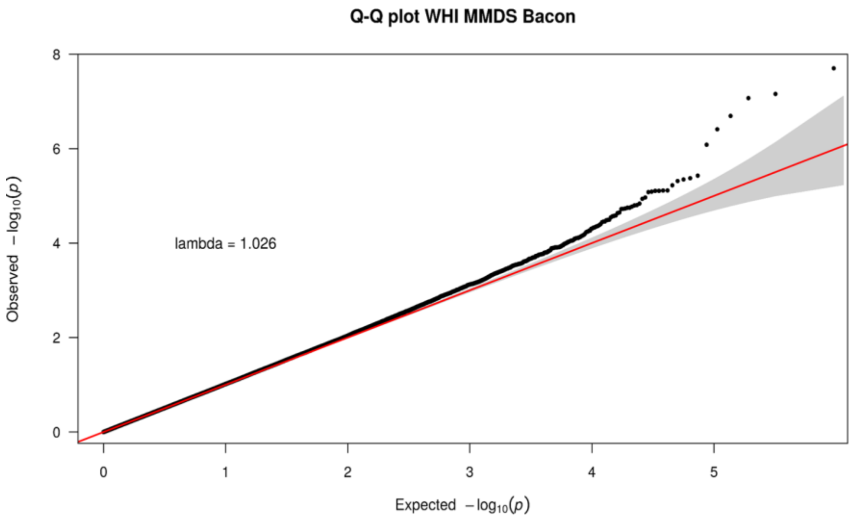
Q-Q plot WHI MMDS Bacon
lambda = 1.026
Observed $-\log_{10}(p)$
Expected $-\log_{10}(p)$
0
2
4
6
8
0
1
2
3
4
5

REGICOR
450K

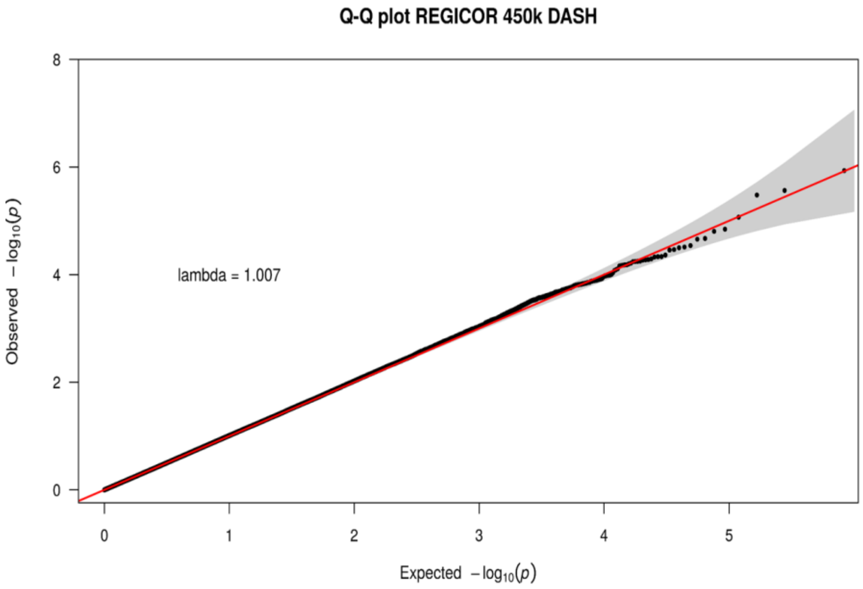

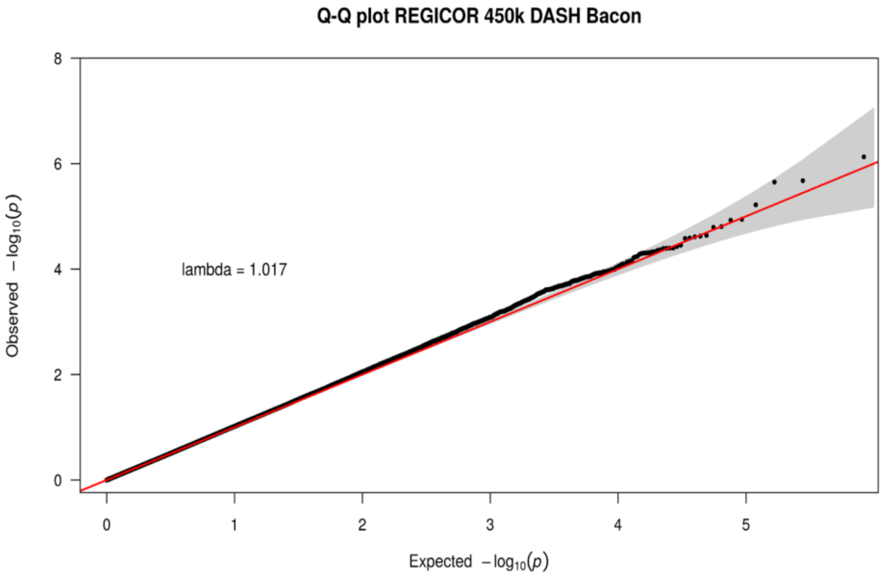

REGICOR
450K

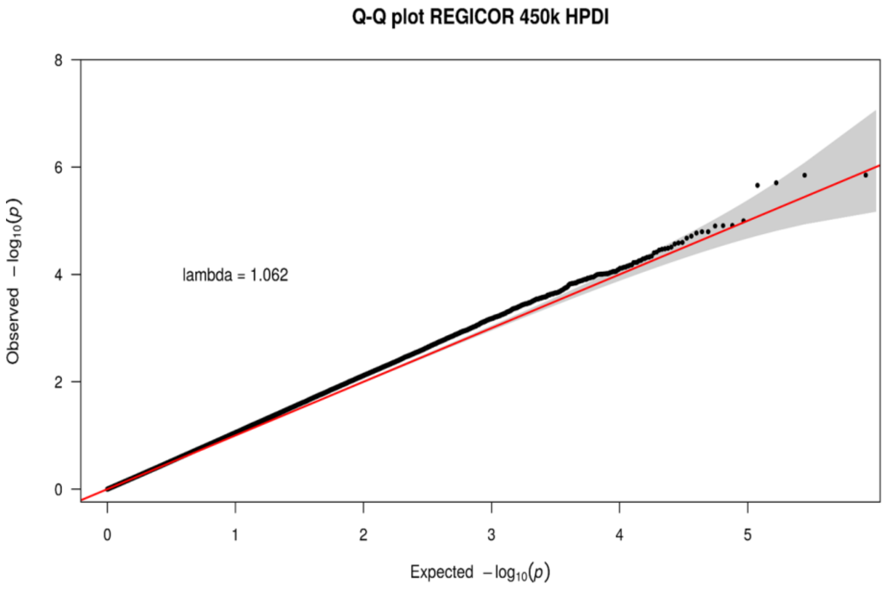

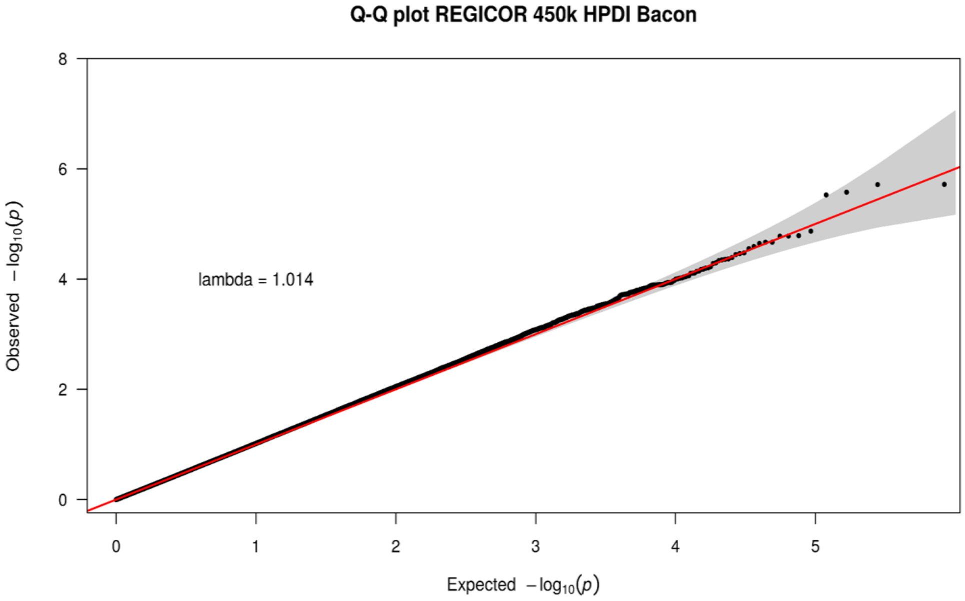

REGICOR
450K

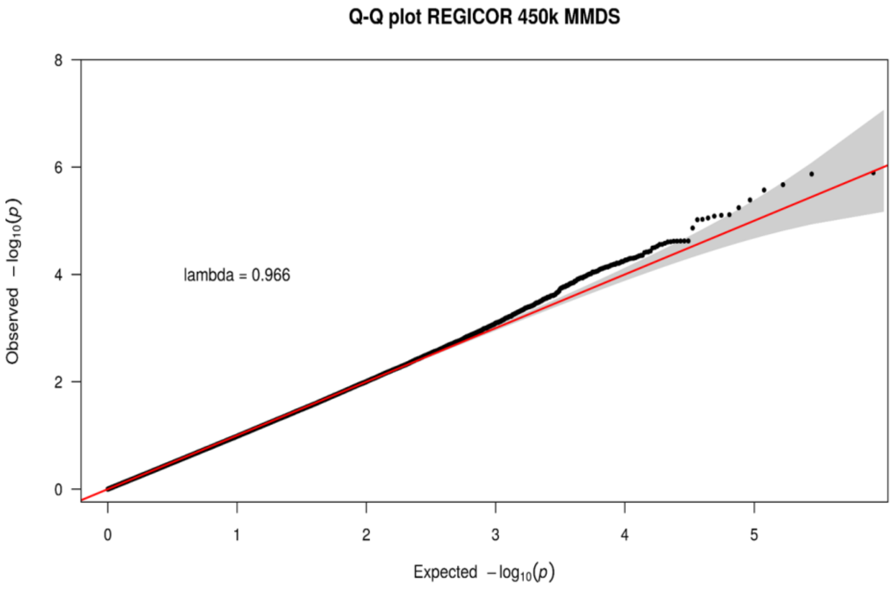

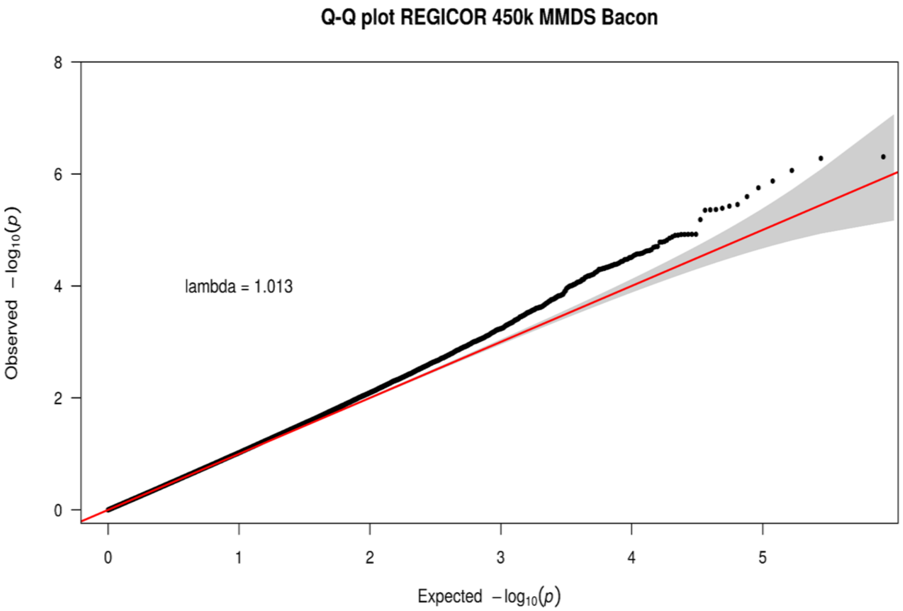

REGICOR
EPIC

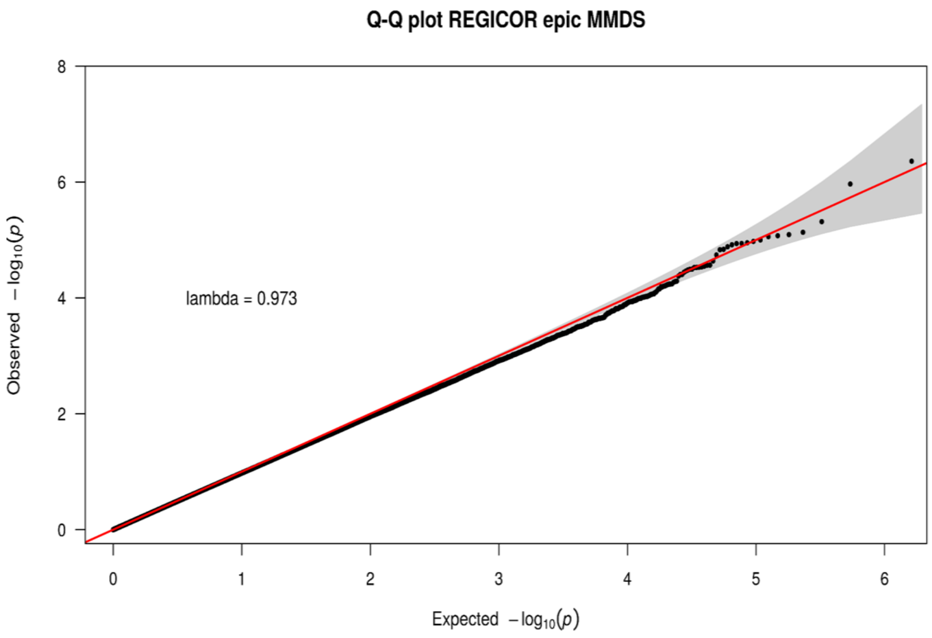

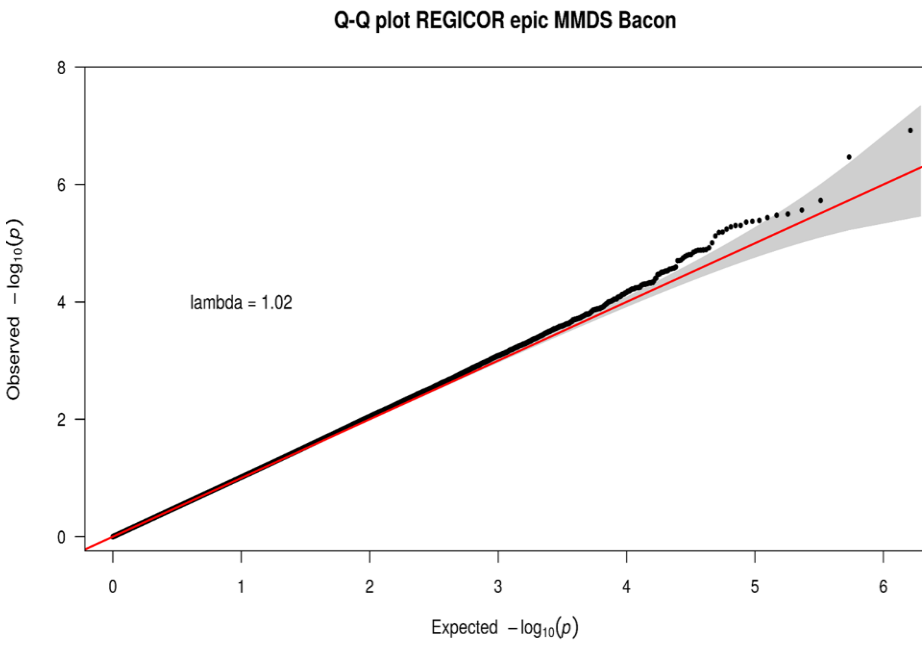

REGICOR
EPIC

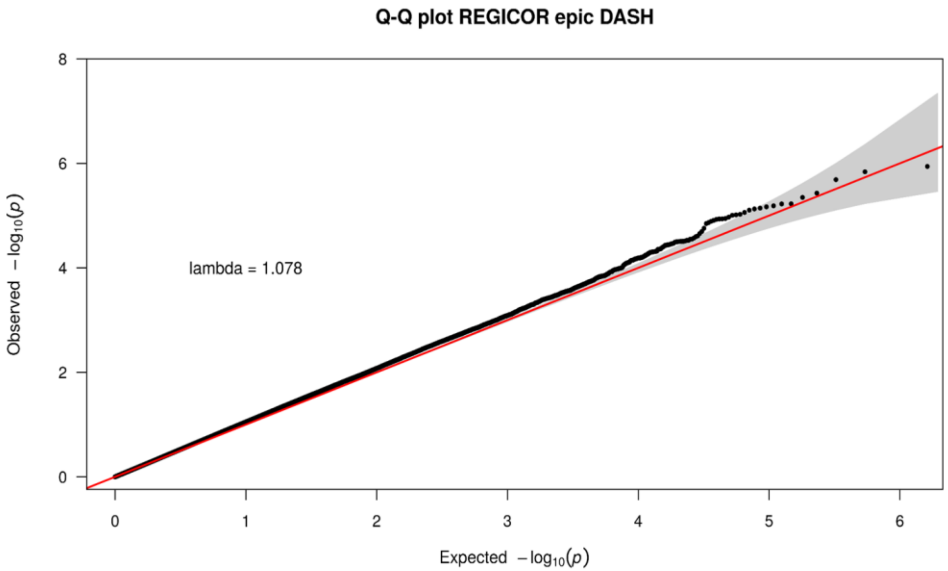

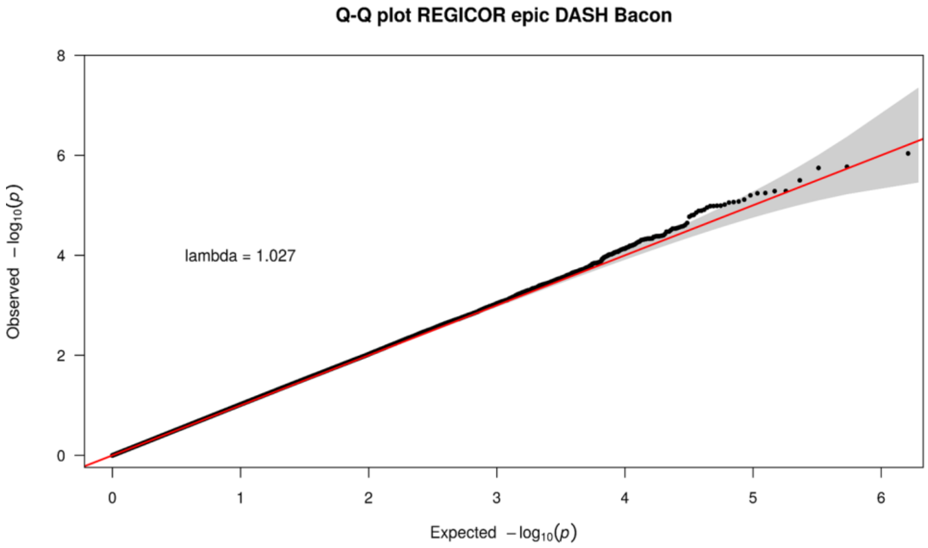

REGICOR
EPIC

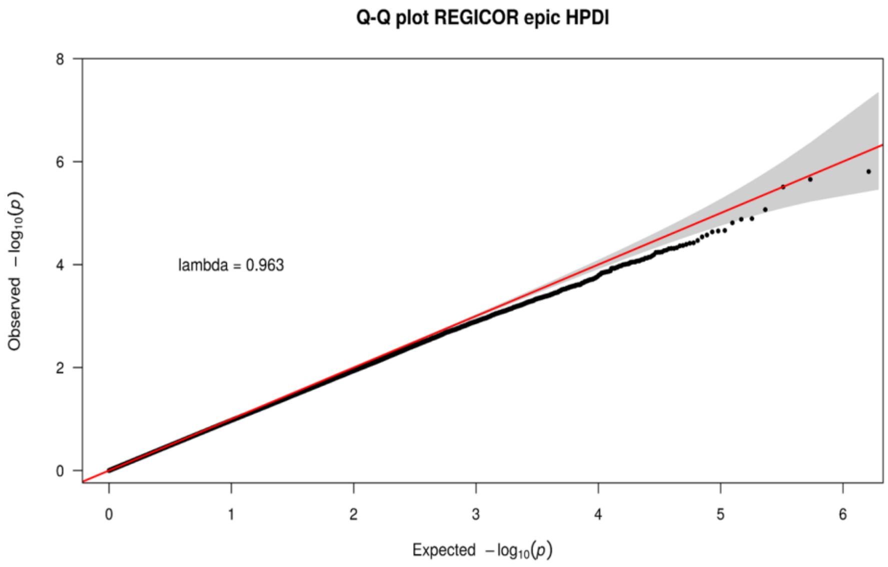

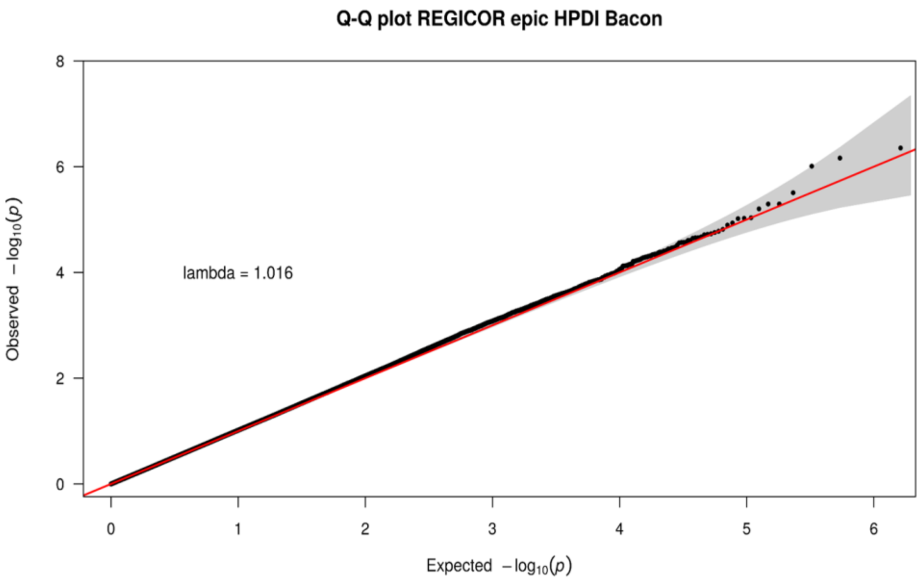

Figure S3: Manhattan plots in each cohort, before and after correction using *bacon* (Pages 17-26)

AIRWAVE

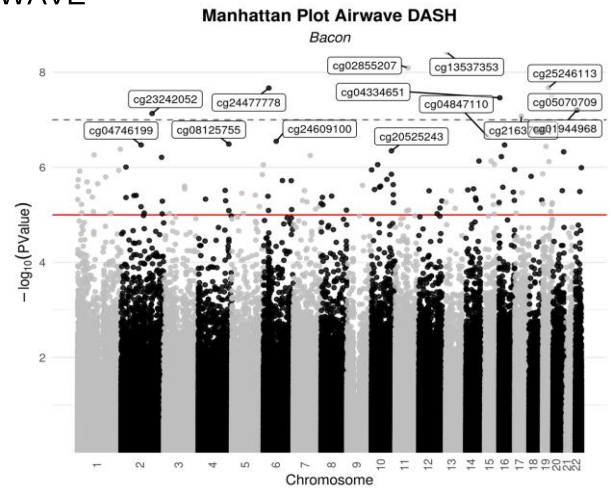

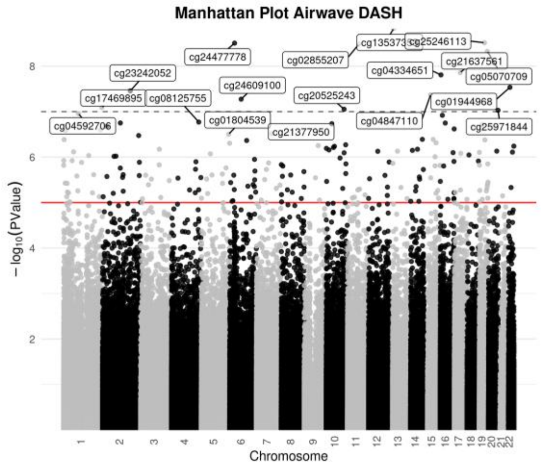

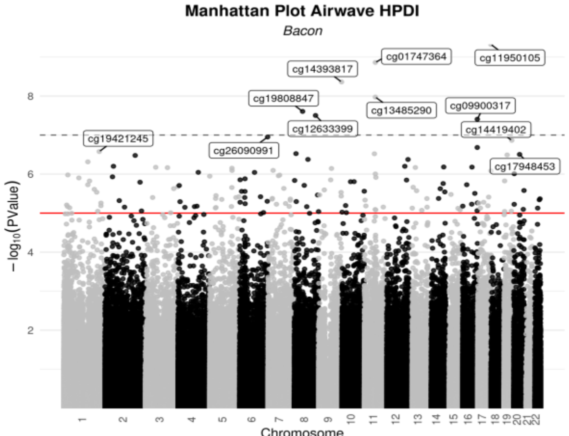

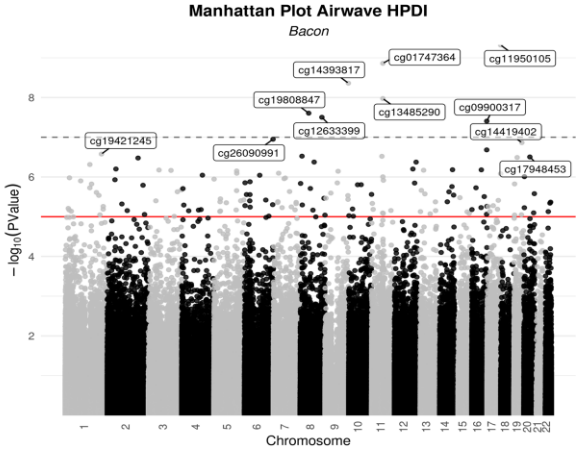

AIRWAVE

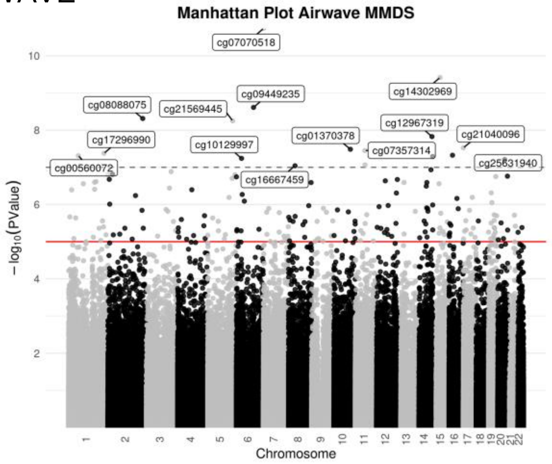
Manhattan Plot Airwave MMDS
cg07070518
cg09449235
cg14302969
cg08088075
cg21569445
cg12967319
cg17296990
cg10129997
cg01370378
cg21040096
cg07357314
cg00560072
cg25631940
cg16667459
− log10(PValue)
Chromosome

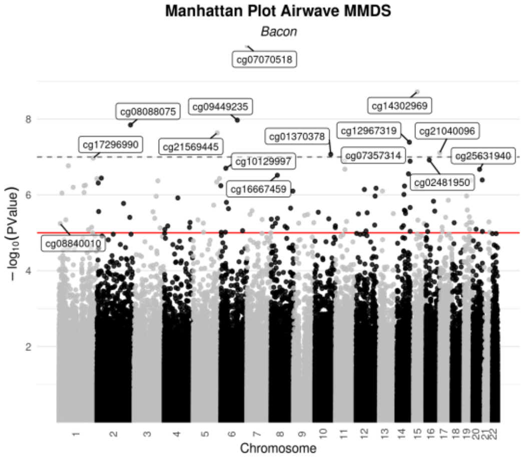
Manhattan Plot Airwave MMDS
Bacon
cg07070518
cg08088075
cg09449235
cg14302969
cg01370378
cg12967319
cg21040096
cg17296990
cg21569445
cg07357314
cg25631940
cg10129997
cg02481950
cg16667459
cg08840010
− log10(PValue)
Chromosome

FOS

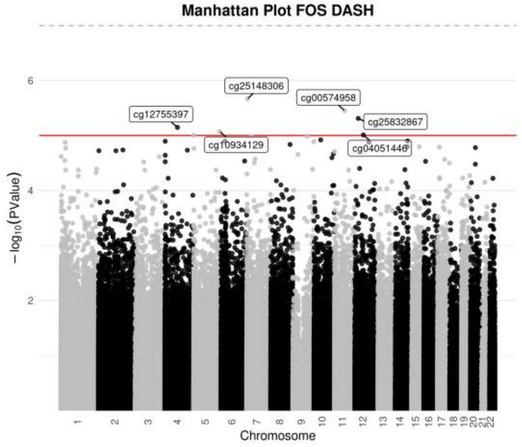
Manhattan Plot FOS DASH
cg25148306
cg00574958
cg12755397
cg25832867
cg10934129
cg04051446
− log10(PValue)
Chromosome

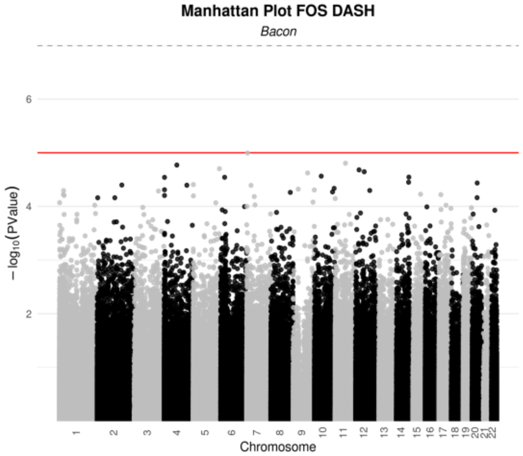
Manhattan Plot FOS DASH
Bacon
− log10(PValue)
Chromosome

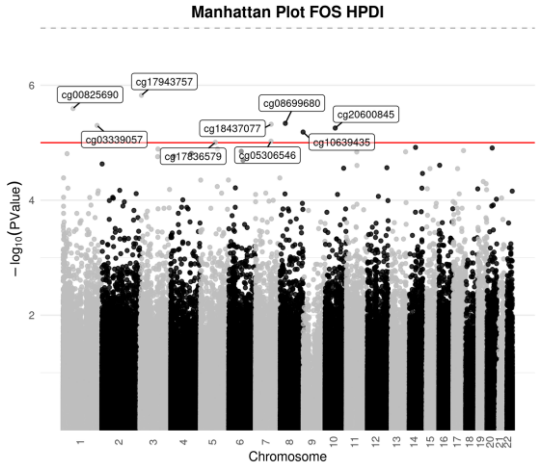
Manhattan Plot FOS HPDI
cg17943757
cg00825690
cg08699680
cg20600845
cg18437077
cg03339057
cg10639435
cg05306546
− log10(PValue)
Chromosome

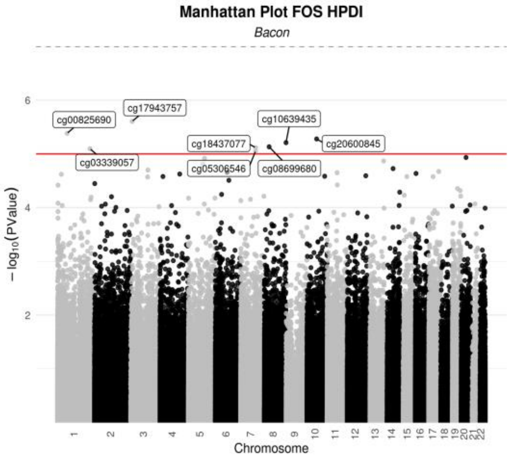
Manhattan Plot FOS HPDI
Bacon
cg17943757
cg00825690
cg10639435
cg18437077
cg20600845
cg03339057
cg05306546
cg08699680
− log10(PValue)
Chromosome

FOS

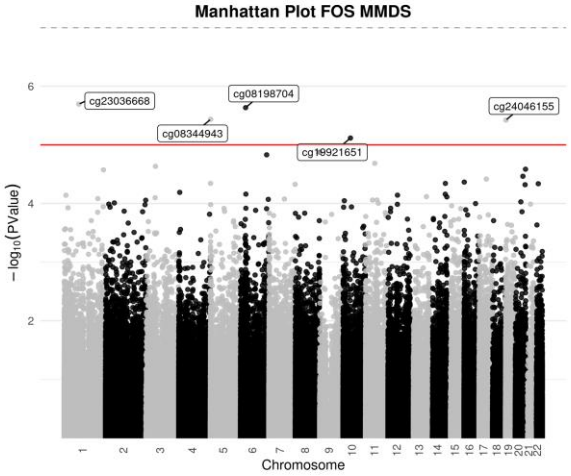
Manhattan Plot FOS MMDS
cg23036668
cg08198704
cg24046155
cg08344943
cg19921651
−log10(PValue)
Chromosome

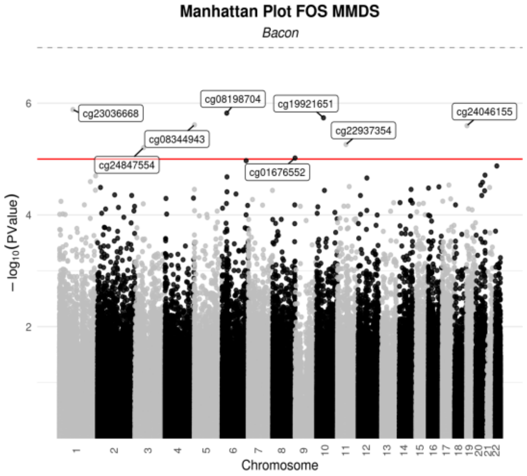
Manhattan Plot FOS MMDS
Bacon
cg23036668
cg08198704
cg19921651
cg24046155
cg22937354
cg08344943
cg24847554
cg01676552
−log10(PValue)
Chromosome

WHI

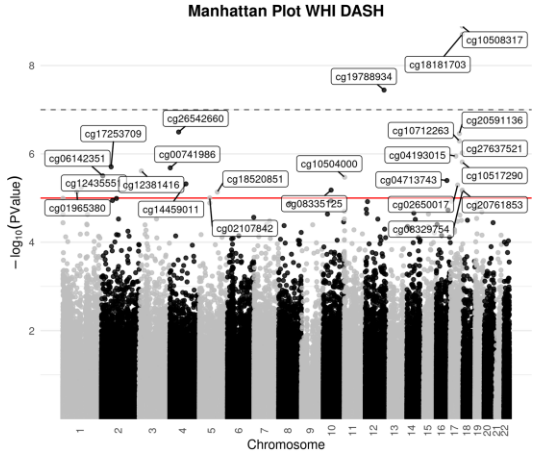
Manhattan Plot WHI DASH
cg10508317
cg18181703
cg19788934
cg26542660
cg17253709
cg06142351
cg00741986
cg12435551
cg12381416
cg01965380
cg14459011
cg18520851
cg10504000
cg10712263
cg20591136
cg04193015
cg27637521
cg04713743
cg10517290
cg08335125
cg02650017
cg20761853
cg02107842
cg08329754
$-\log_{10}(PValue)$
Chromosome

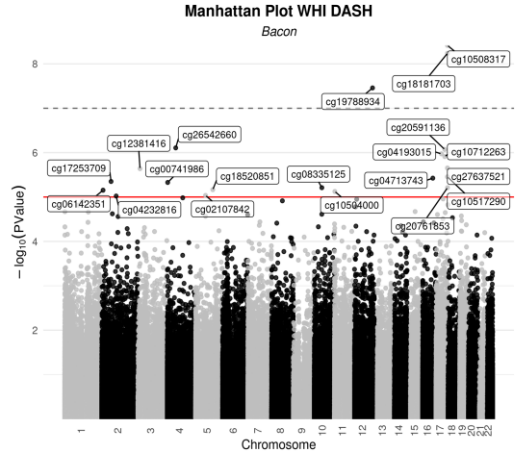
Manhattan Plot WHI DASH
Bacon
cg10508317
cg18181703
cg19788934
cg20591136
cg26542660
cg12381416
cg04193015
cg10712263
cg17253709
cg00741986
cg18520851
cg08335125
cg04713743
cg27637521
cg06142351
cg04232816
cg02107842
cg10504000
cg10517290
cg20761853
$-\log_{10}(PValue)$
Chromosome

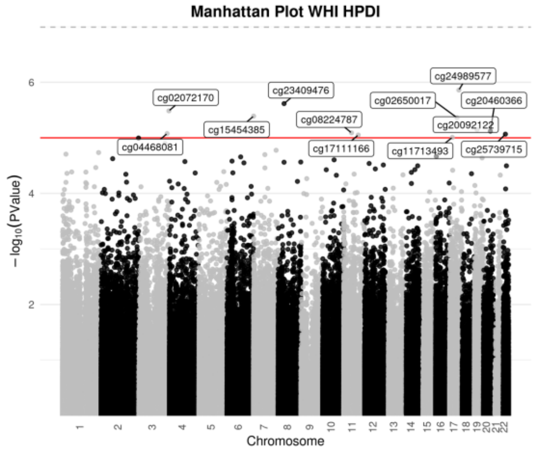
Manhattan Plot WHI HPDI
cg24989577
cg02072170
cg23409476
cg02650017
cg20460366
cg08224787
cg15454385
cg20092122
cg04468081
cg17111166
cg11713493
cg25739715
$-\log_{10}(PValue)$
Chromosome

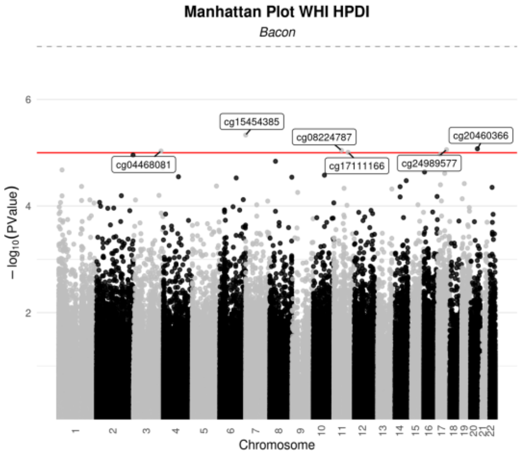
Manhattan Plot WHI HPDI
Bacon
cg15454385
cg08224787
cg20460366
cg04468081
cg17111166
cg24989577
$-\log_{10}(PValue)$
Chromosome

WHI

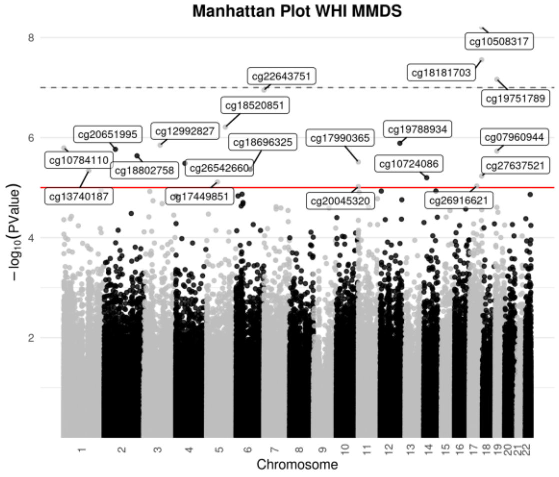
Manhattan Plot WHI MMDS
cg10508317
cg22643751
cg18181703
cg19751789
cg18520851
cg20651995
cg12992827
cg19788934
cg18696325
cg17990365
cg07960944
cg10784110
cg18802758
cg26542660
cg10724086
cg27637521
cg13740187
cg17449851
cg20045320
cg26916621
− log10(PValue)
Chromosome

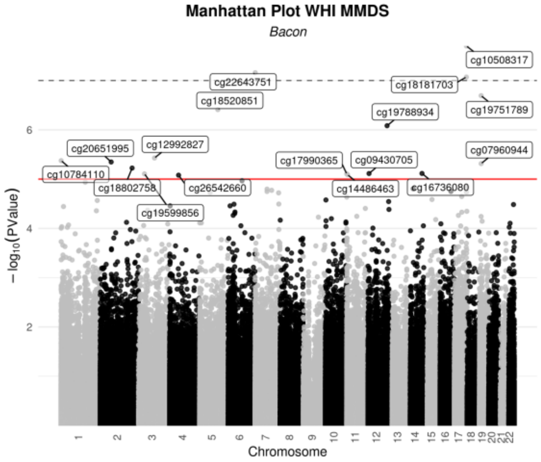
Manhattan Plot WHI MMDS
Bacon
cg10508317
cg22643751
cg18181703
cg18520851
cg19788934
cg19751789
cg20651995
cg12992827
cg07960944
cg17990365
cg09430705
cg10784110
cg18802758
cg26542660
cg14486463
cg16736080
cg19599856
− log10(PValue)
Chromosome

REGICOR
450K

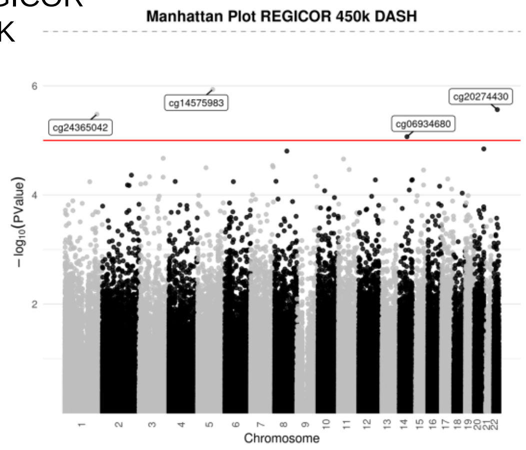

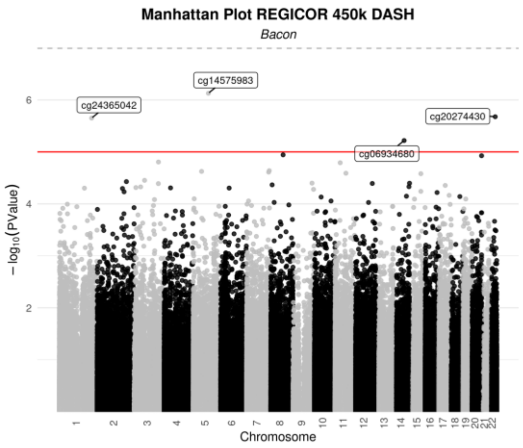

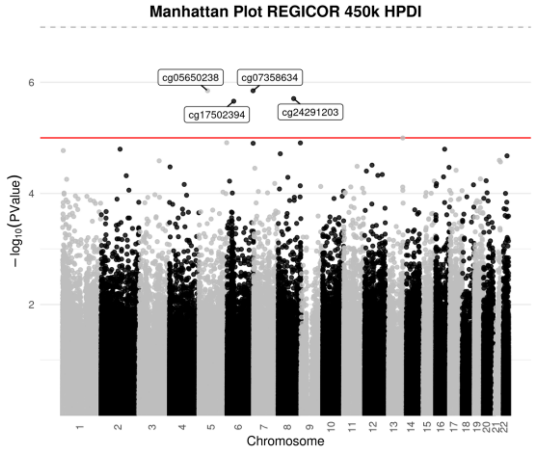

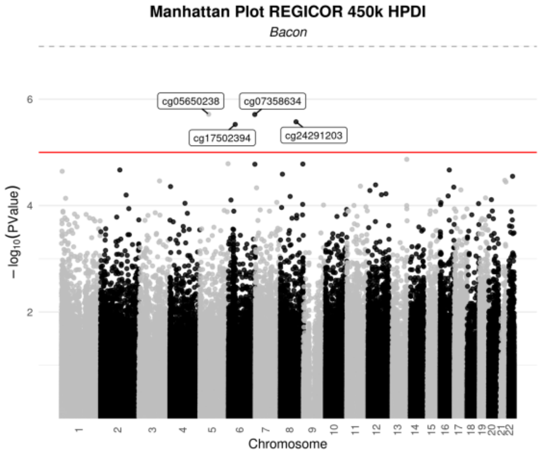

REGICOR 450K

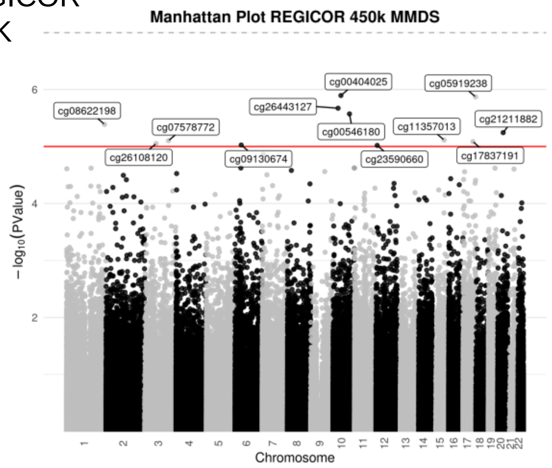

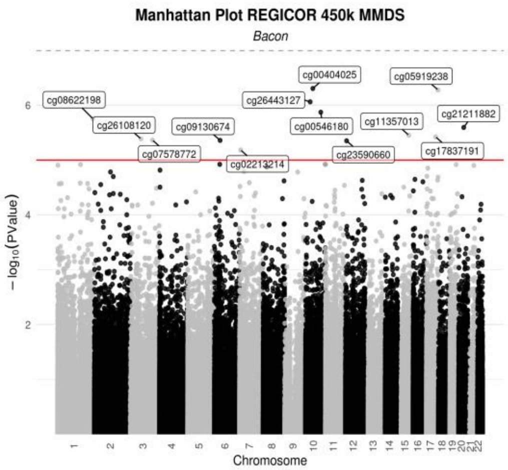

REGICOR
EPIC

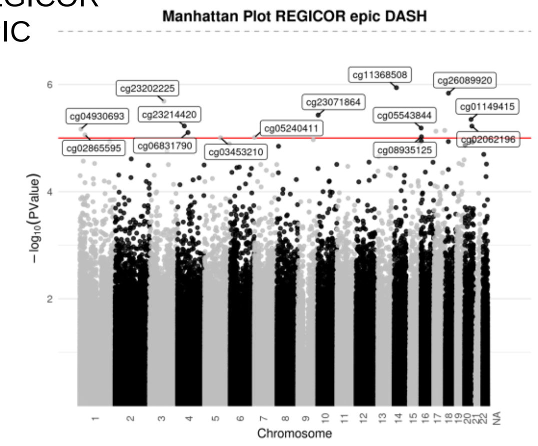

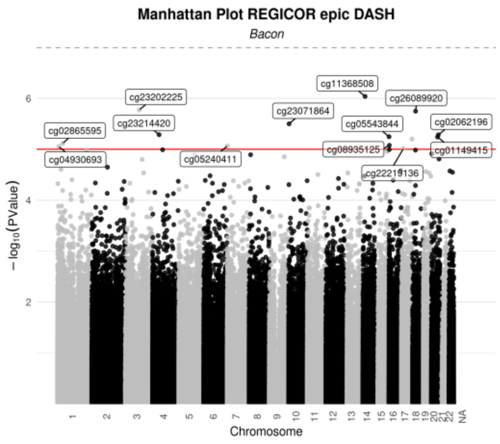

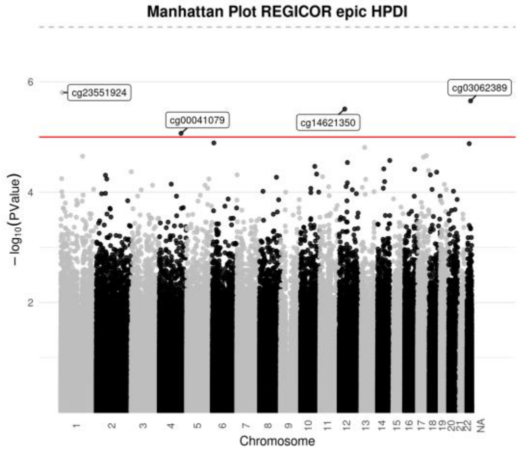

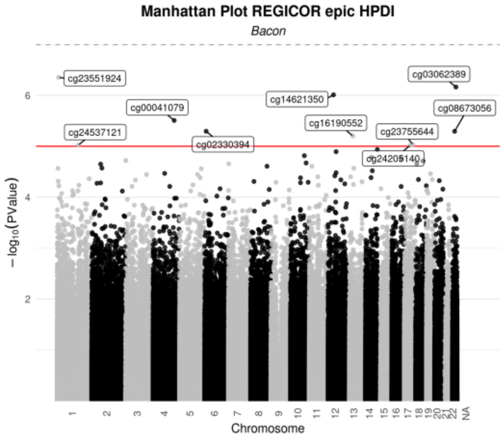

REGICOR
EPIC

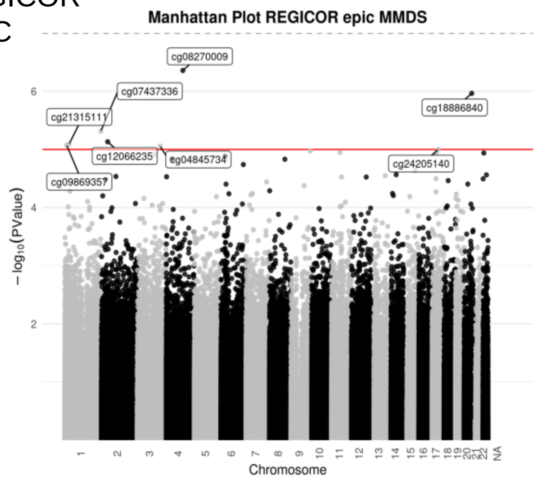

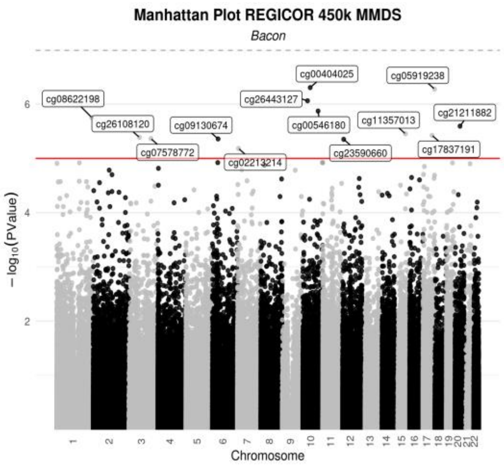

Figure S4: Pearson's correlation coefficients between diet quality scores in the 4 cohorts (1/2)

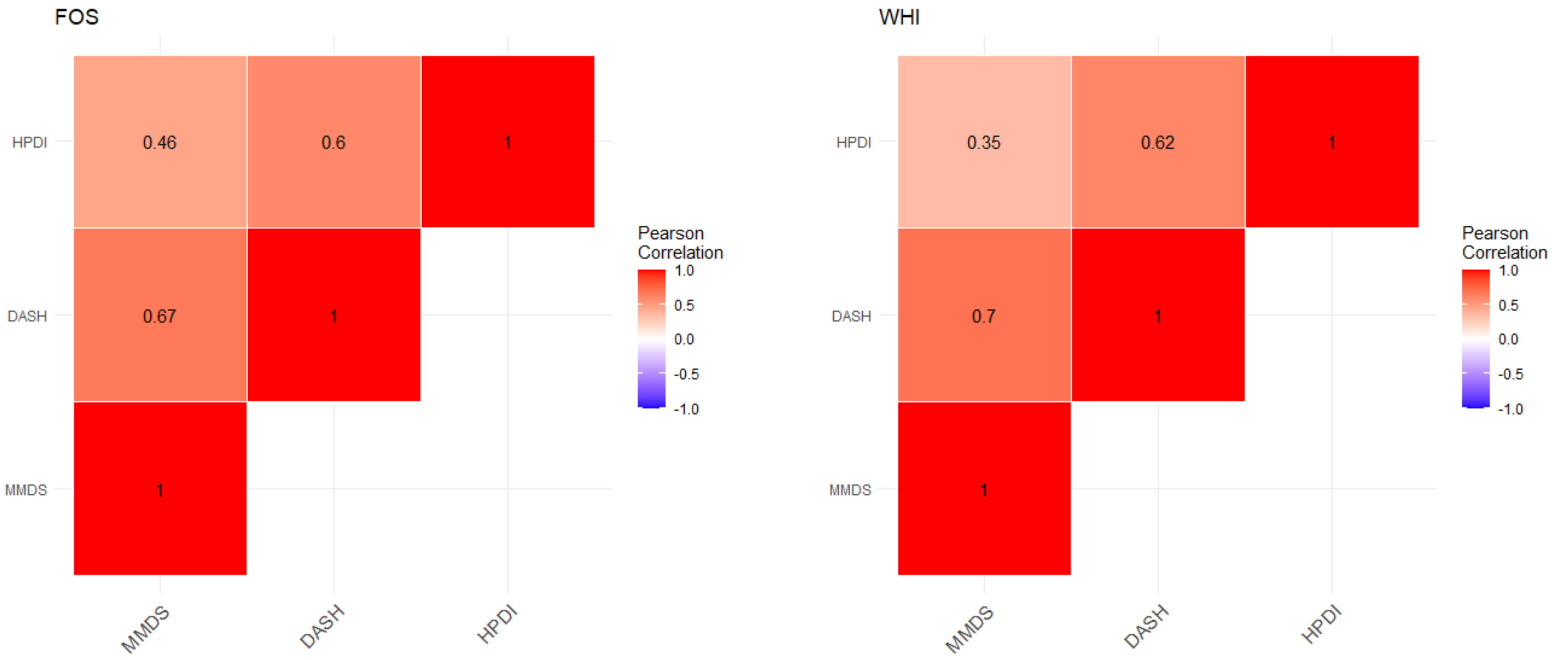

Figure S4: Pearson's correlation coefficients between diet quality scores in the 4 cohorts (2/2)

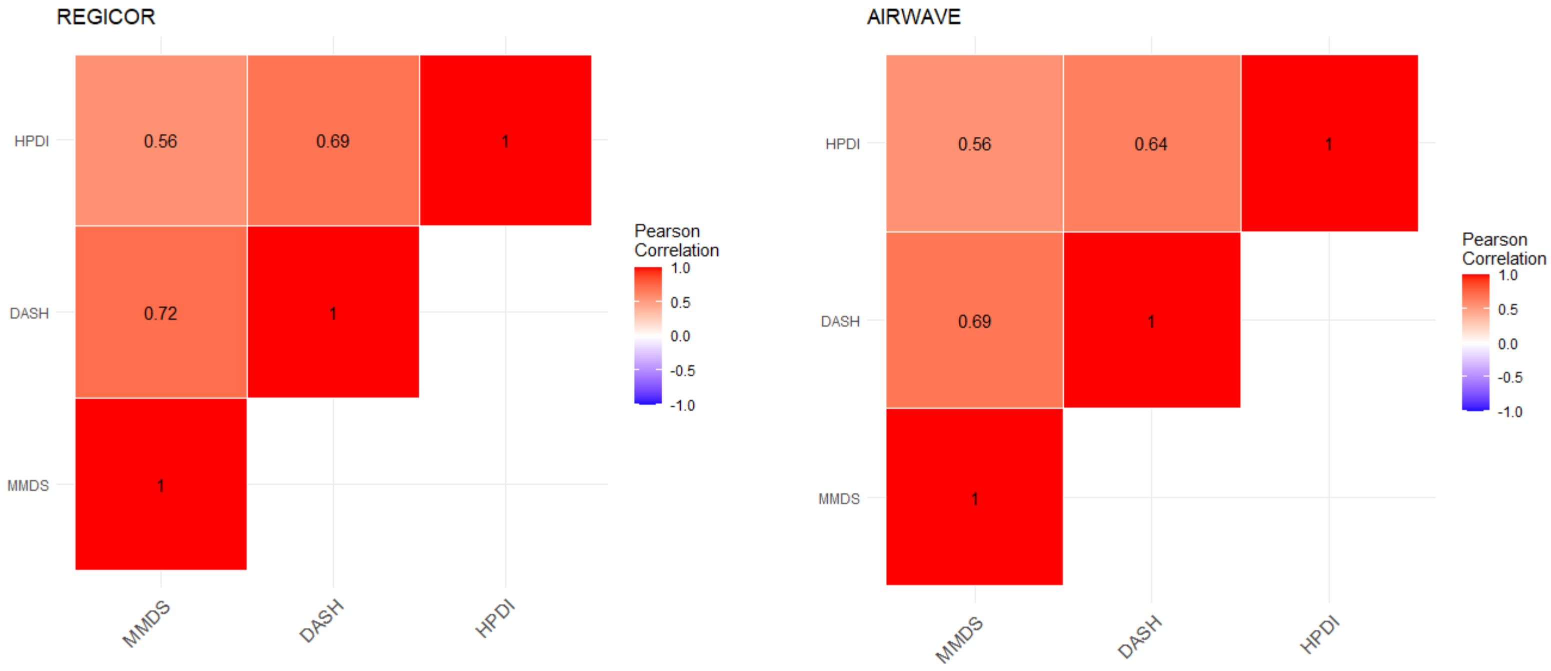

Figure S5: Forest plots for the association of DNA methylation and diet quality for Bonferroni-corrected significant CpGs in each sample. The blue line is the combined estimate (pages 29 to 37)

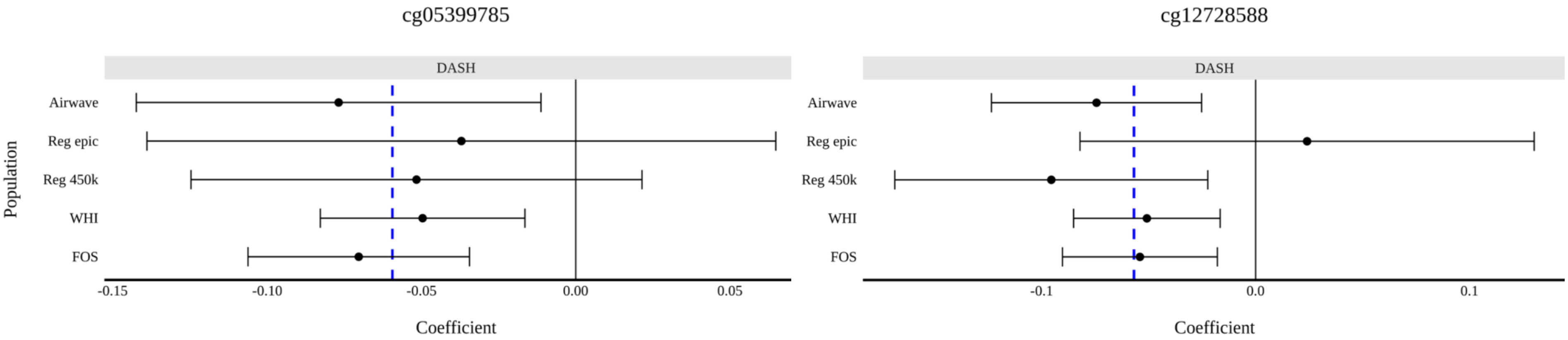

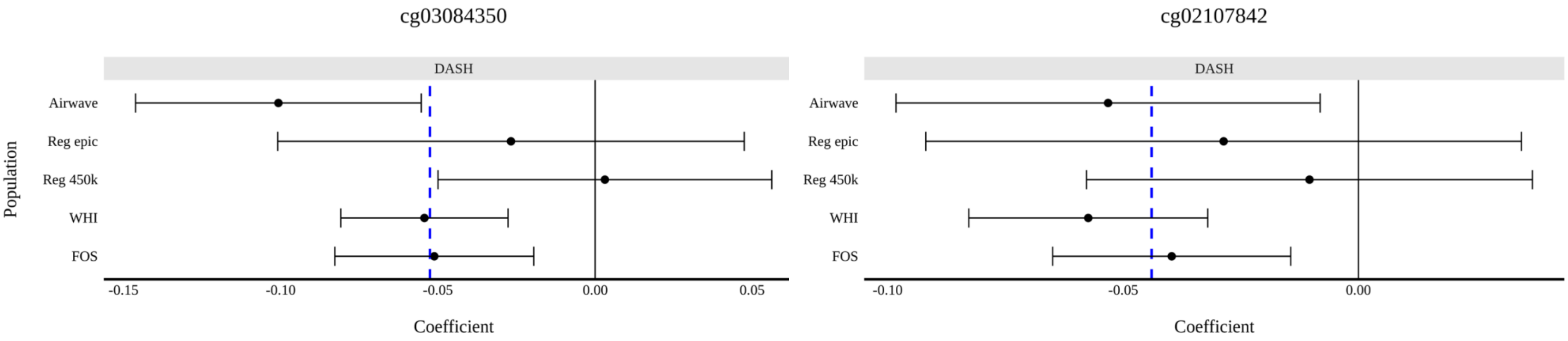
cg03084350
DASH
Airwave
Reg epic
Reg 450k
WHI
FOS
Population
-0.15
-0.10
-0.05
0.00
0.05
Coefficient
cg02107842
DASH
Airwave
Reg epic
Reg 450k
WHI
FOS
-0.10
-0.05
0.00
Coefficient

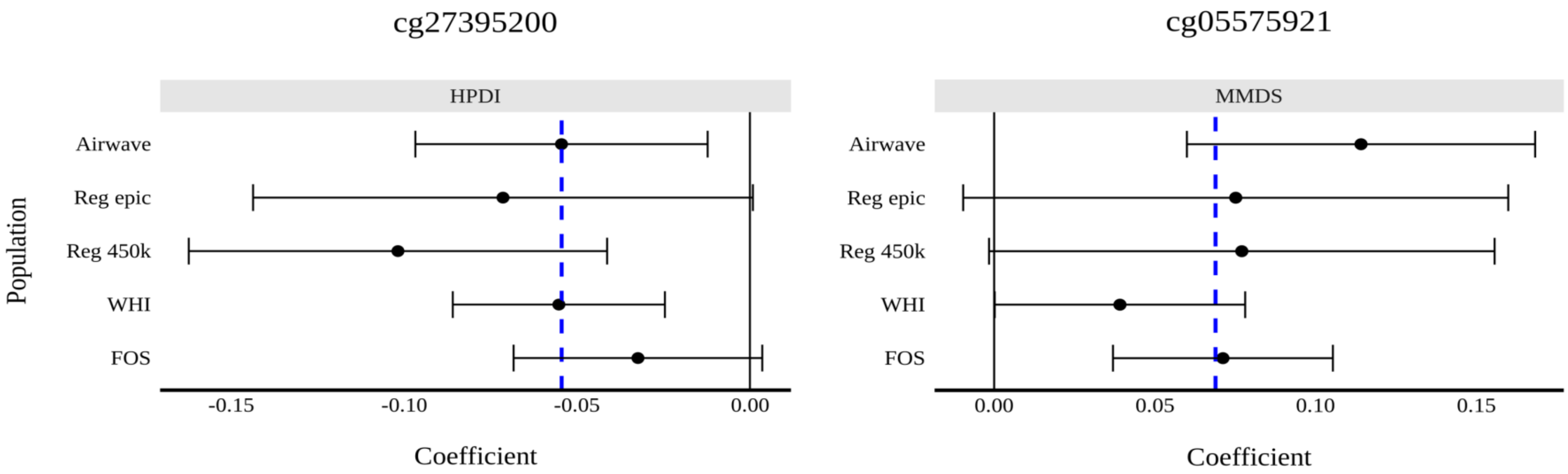
cg27395200
HPDI
Airwave
Reg epic
Reg 450k
WHI
FOS
Population
-0.15
-0.10
-0.05
0.00
Coefficient
cg05575921
MMDS
Airwave
Reg epic
Reg 450k
WHI
FOS
0.00
0.05
0.10
0.15
Coefficient

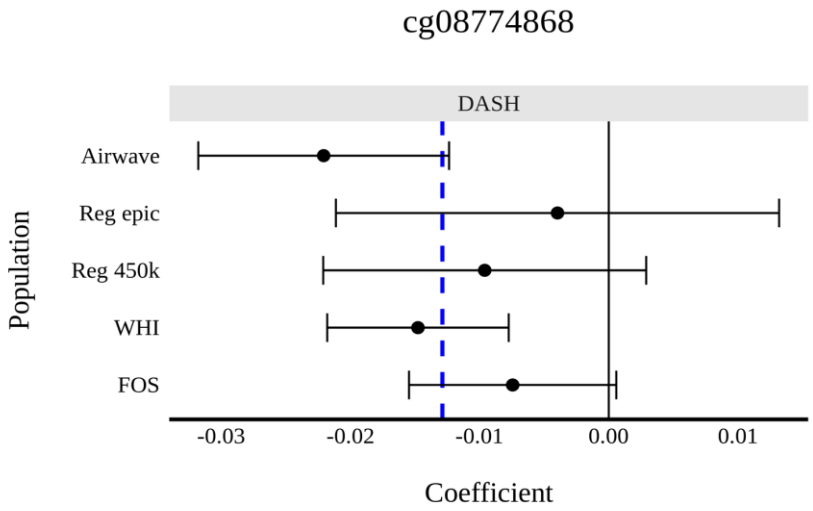
cg08774868
DASH
Population
Airwave
Reg epic
Reg 450k
WHI
FOS
-0.03
-0.02
-0.01
0.00
0.01
Coefficient

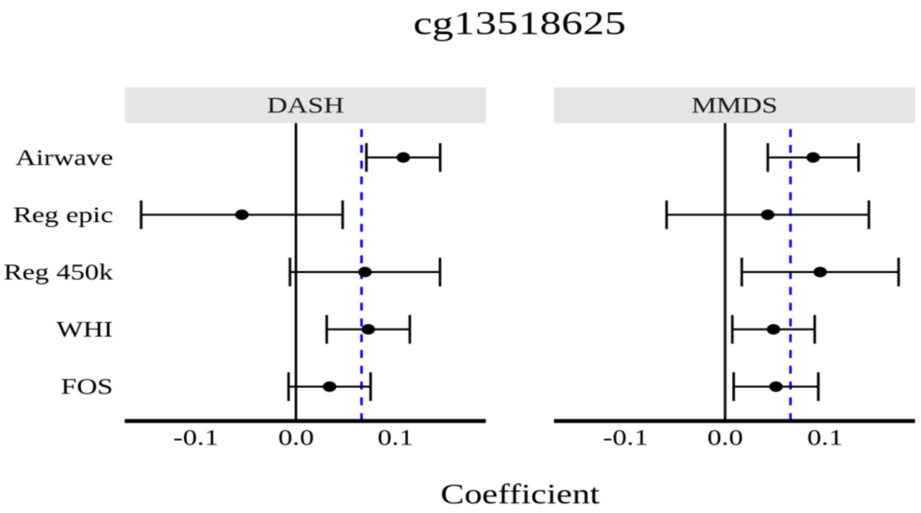
cg13518625
DASH
MMDS
Airwave
Reg epic
Reg 450k
WHI
FOS
-0.1
0.0
0.1
-0.1
0.0
0.1
Coefficient

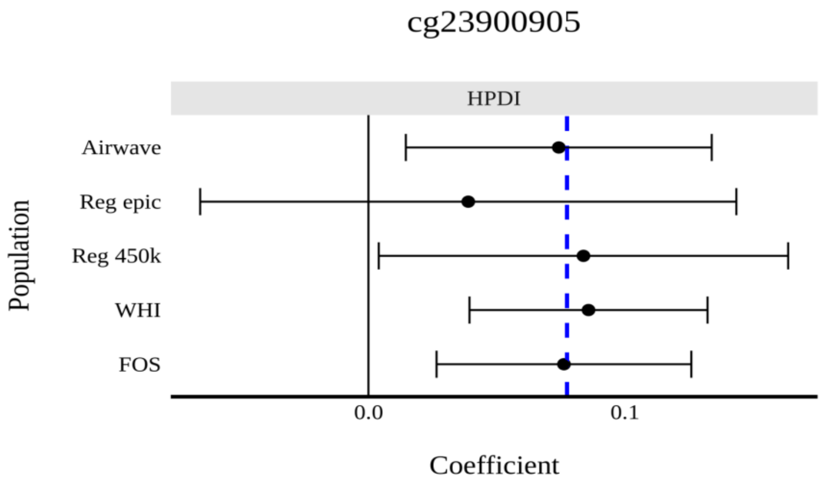
cg23900905
HPDI
Airwave
Reg epic
Reg 450k
WHI
FOS
Population
0.0
0.1
Coefficient

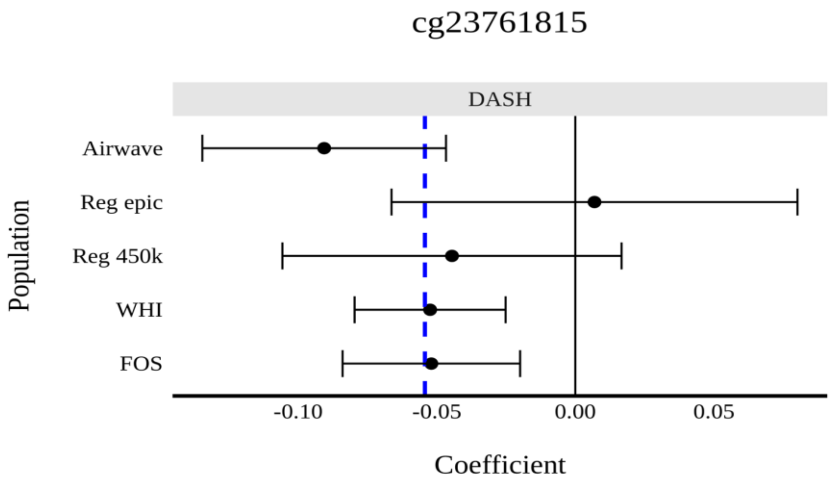
cg23761815
DASH
Airwave
Reg epic
Reg 450k
WHI
FOS
Population
-0.10
-0.05
0.00
0.05
Coefficient

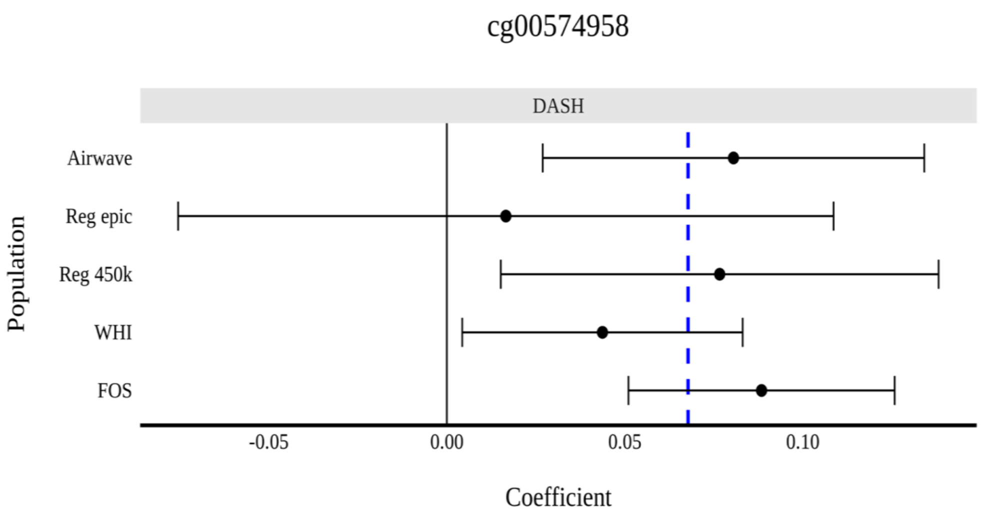
cg00574958
DASH
Population
Airwave
Reg epic
Reg 450k
WHI
FOS
-0.05
0.00
0.05
0.10
Coefficient

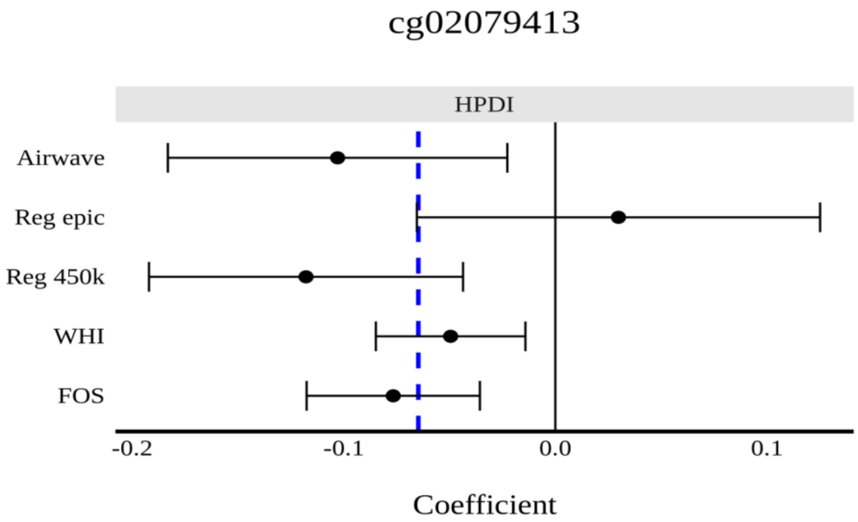
cg02079413
HPDI
Airwave
Reg epic
Reg 450k
WHI
FOS
-0.2
-0.1
0.0
0.1
Coefficient

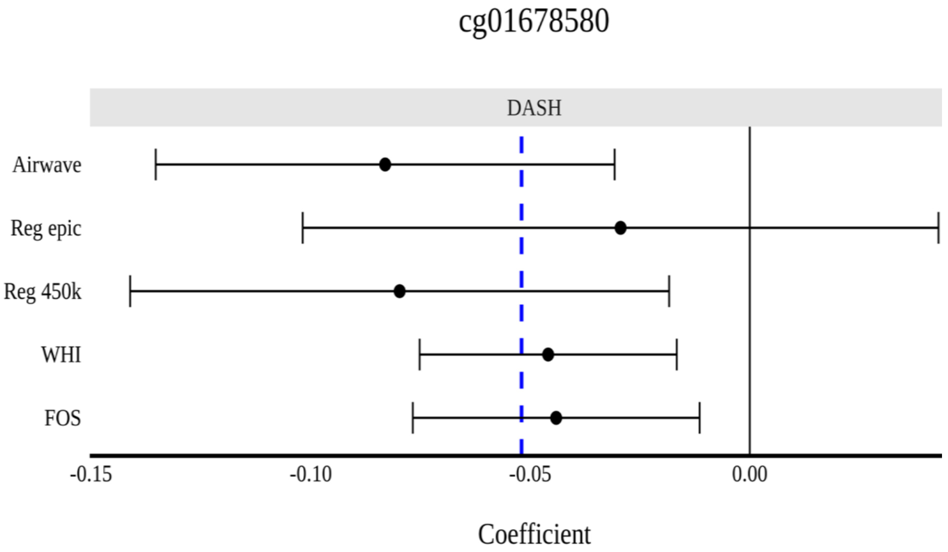
cg01678580
DASH
Airwave
Reg epic
Reg 450k
WHI
FOS
-0.15
-0.10
-0.05
0.00
Coefficient

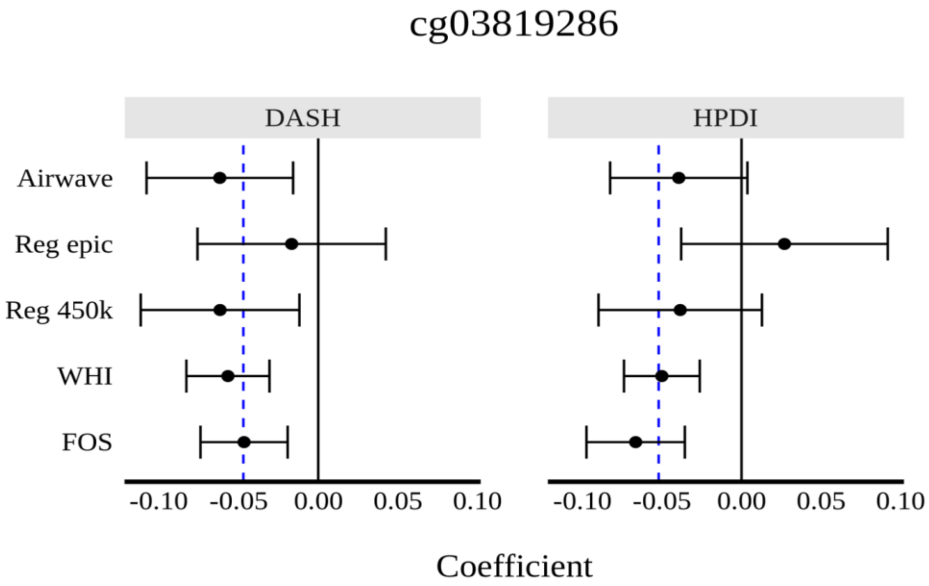
cg03819286
DASH
HPDI
Airwave
Reg epic
Reg 450k
WHI
FOS
-0.10
-0.05
0.00
0.05
0.10
Coefficient

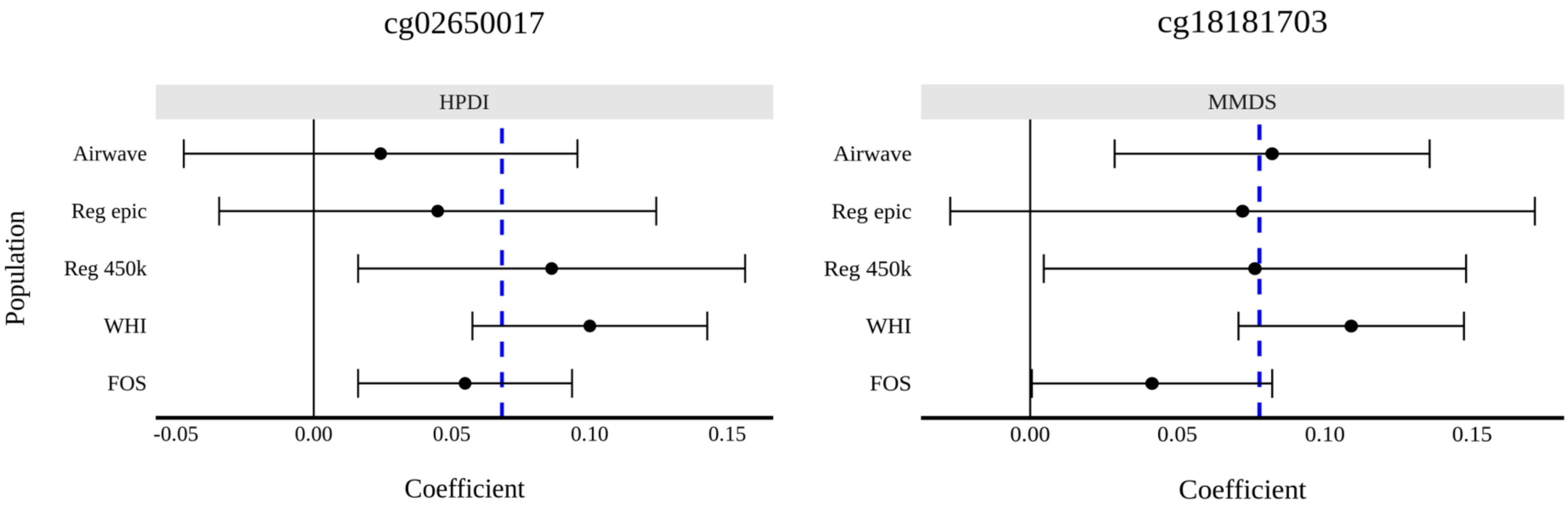

cg02650017
cg18181703
HPDI
MMDS
Population
Airwave
Reg epic
Reg 450k
WHI
FOS
-0.05
0.00
0.05
0.10
0.15
Coefficient

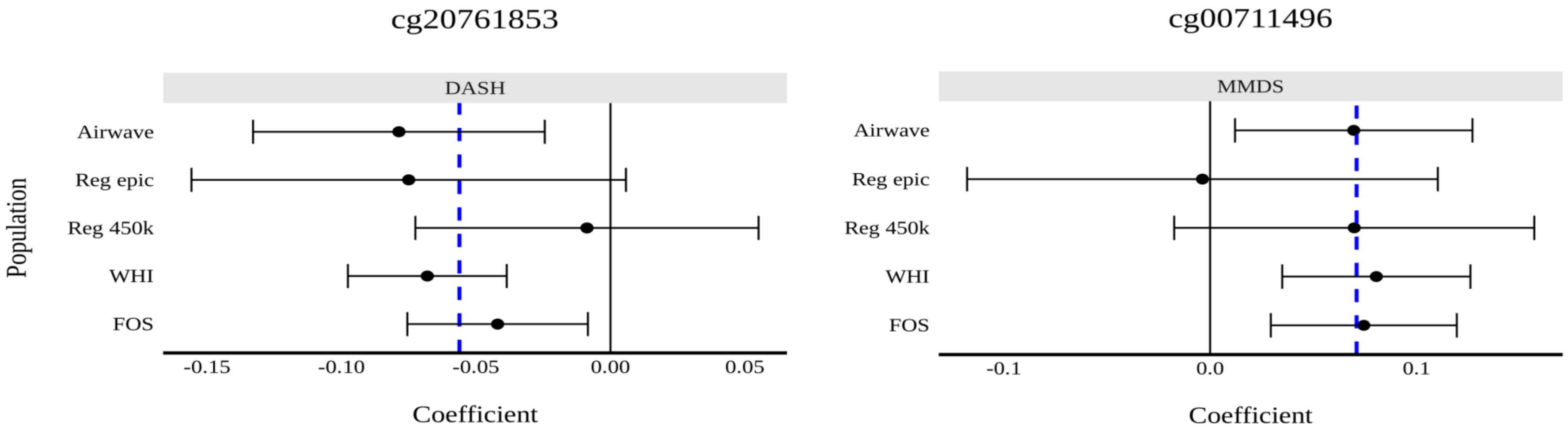
cg20761853
DASH
Airwave
Reg epic
Reg 450k
WHI
FOS
Population
-0.15
-0.10
-0.05
0.00
0.05
Coefficient
cg00711496
MMDS
Airwave
Reg epic
Reg 450k
WHI
FOS
-0.1
0.0
0.1
Coefficient

Figure S6: Correlation of EWAS coefficients of 18 Bonferroni-significant CpGs (p-value<1.08x10^{-7})

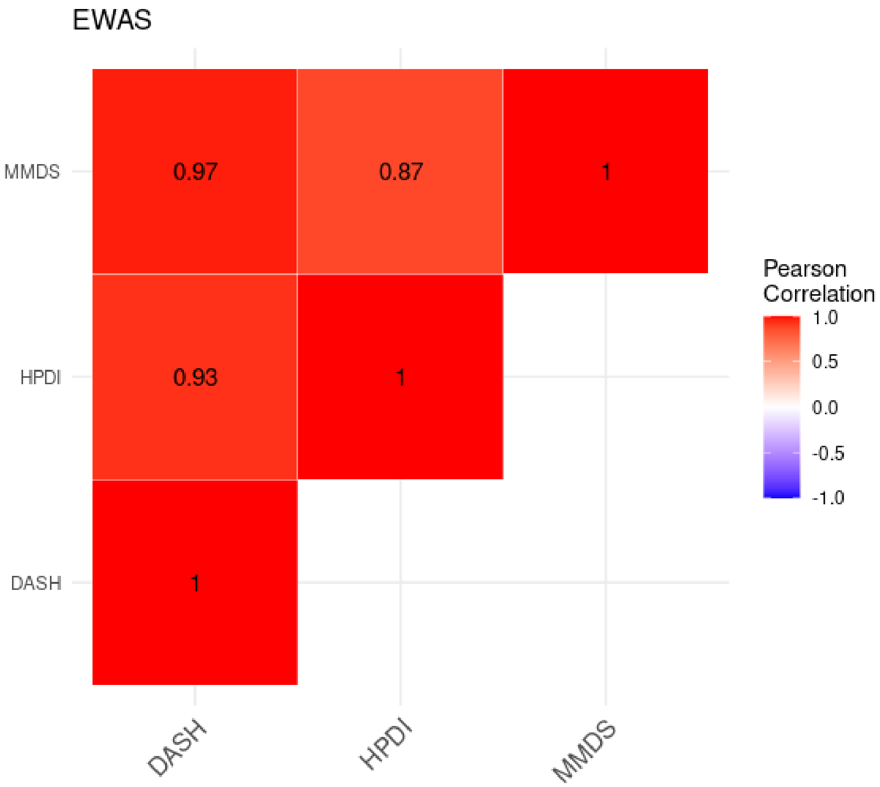

Figure S7: Forest plot for the association of DNA methylation at cg05575921 (*AHRR*) and MMDS diet score in each sample stratified by smoking status.

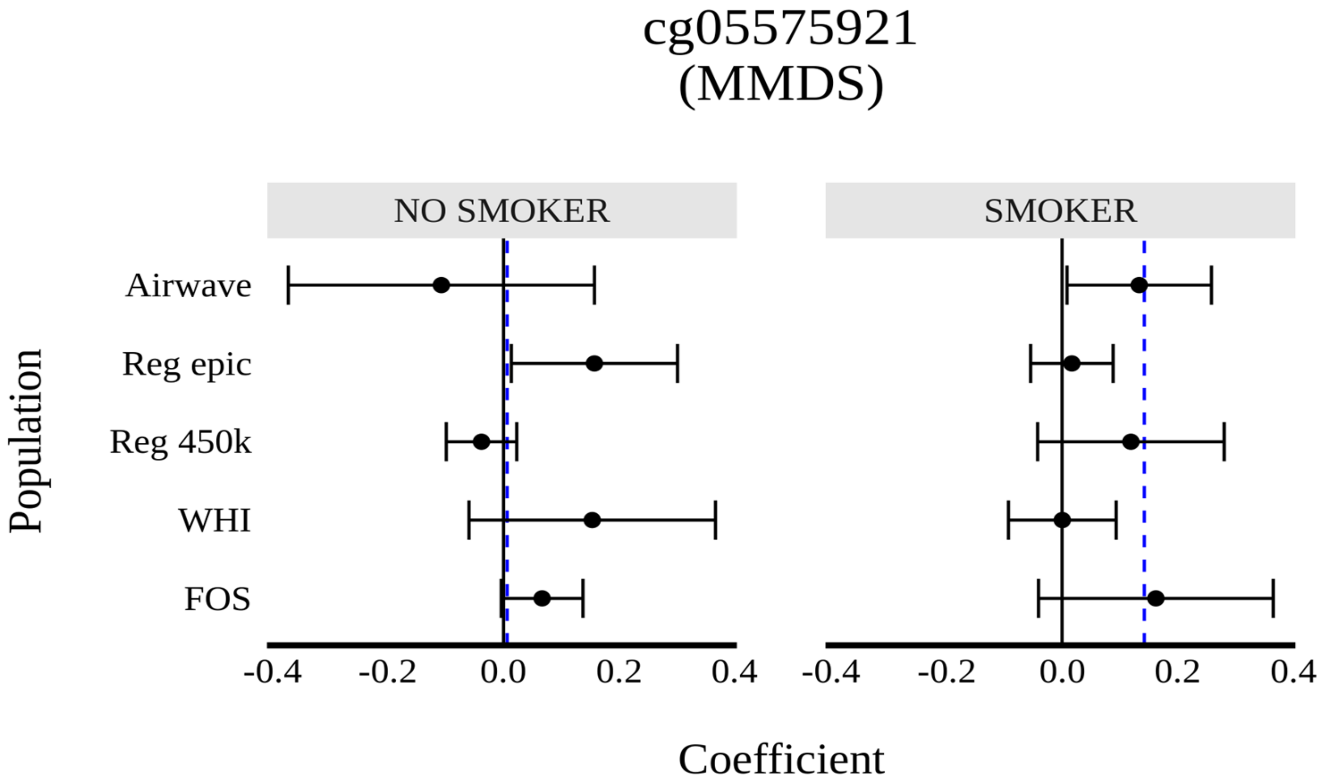

Figure S8: Distribution of diet-associated gene expression across tissues (GTEx database).

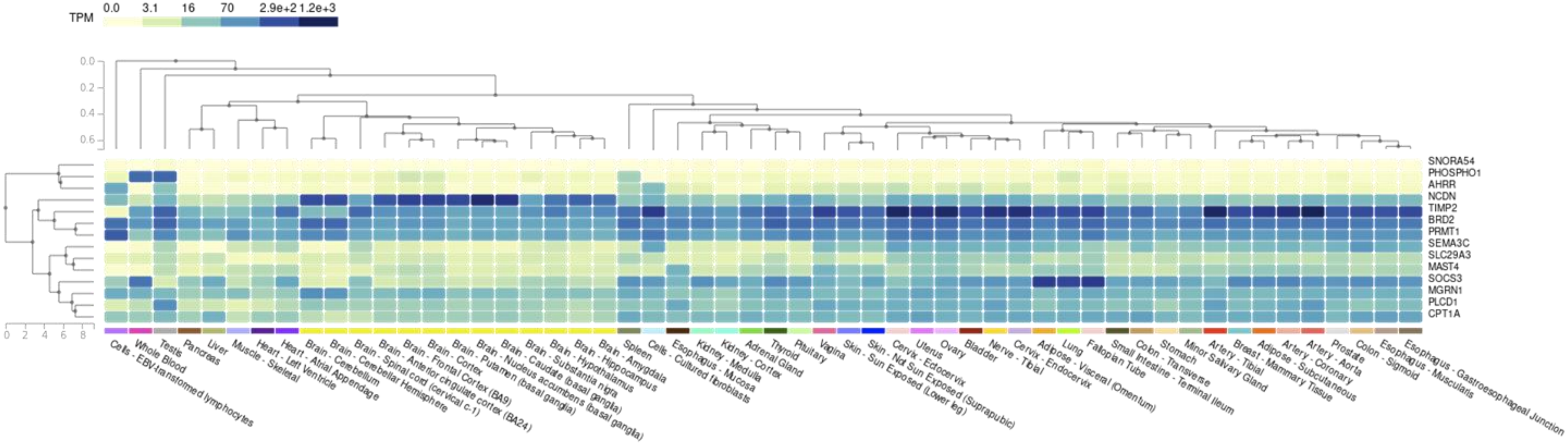

Figure S9: Overrepresentation results for FDR-significant diet-related CpGs annotates to genes for Gene Position (A), CpGs Island Relative Positions (B) and Chromatin State (C)

A

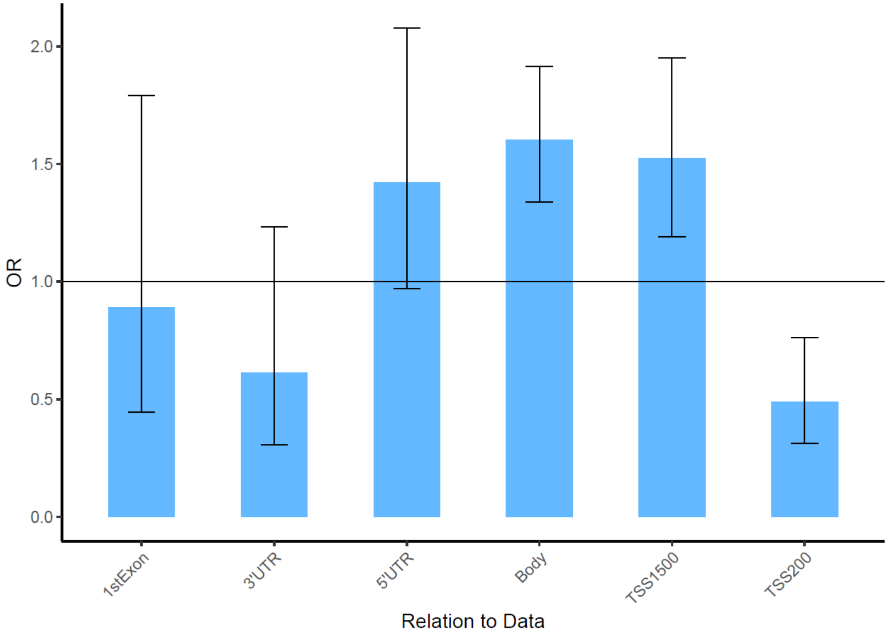

B

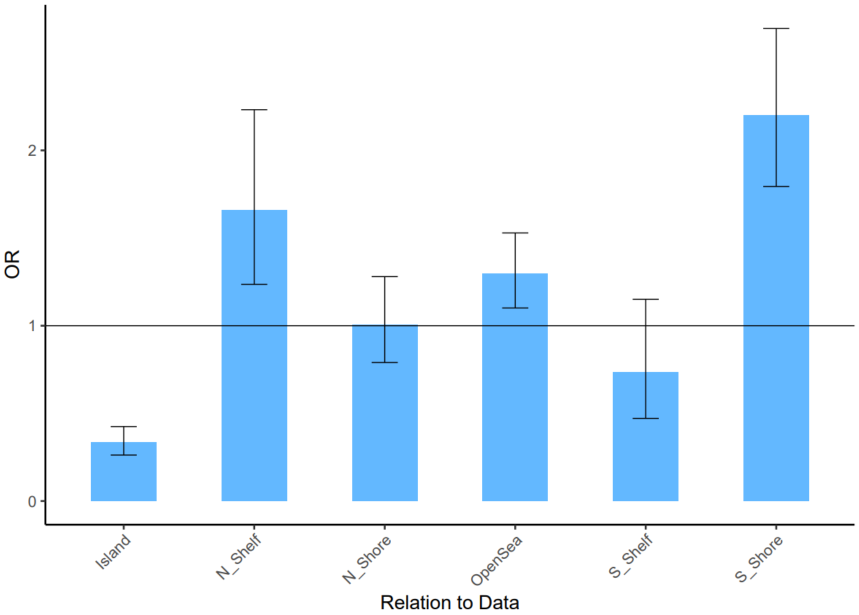

Figure S9: Overrepresentation results for FDR-significant diet-related CpGs annotates to genes for Gene Position (A), CpGs Island Relative Positions (B) and Chromatin State (C)

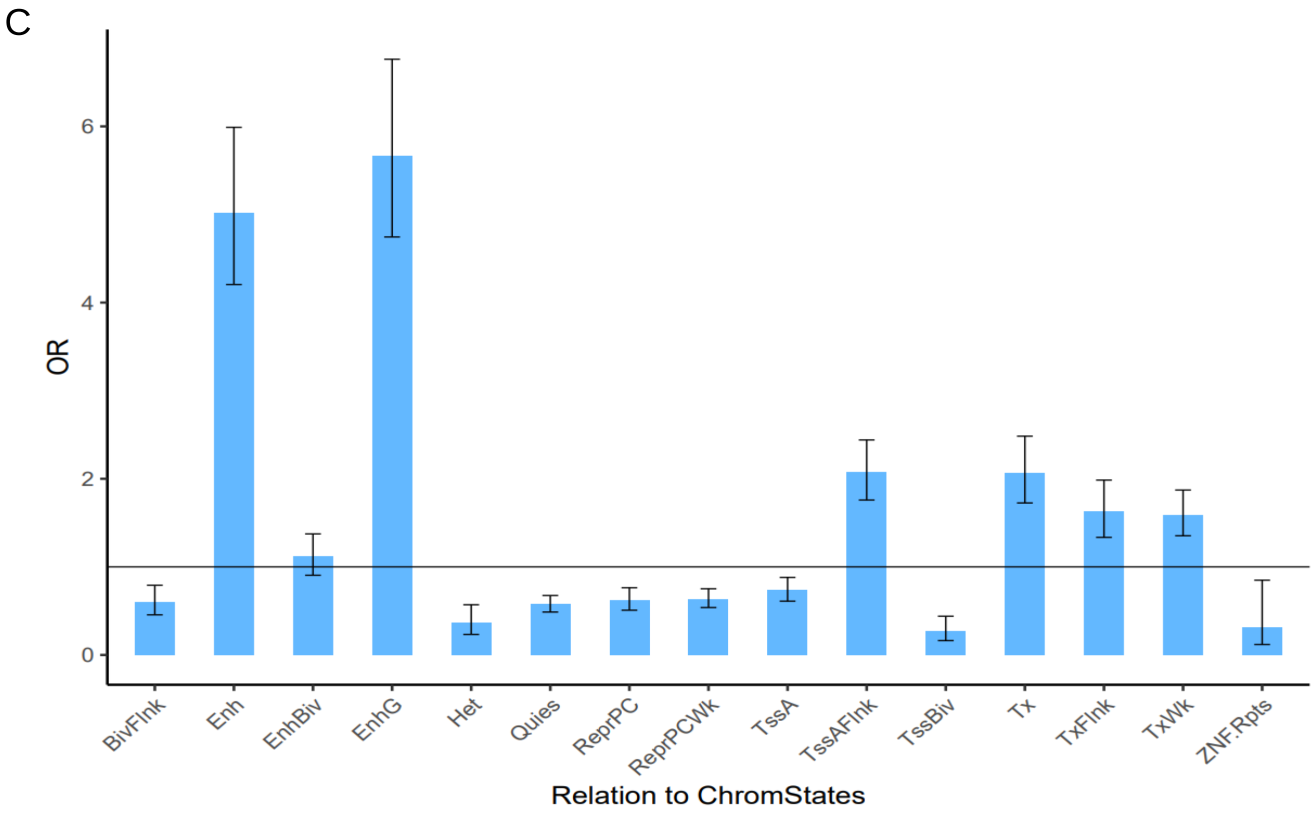

Supplement: zwad317_Supplementary_Data [file zwad317_supplementary_data.zip › Supplemental Figures 040823.pdf]
