# Supplementary material for: Blood DNA methylation signature of diet quality and association with cardiometabolic traits
Source: Eur J Prev Cardiol. 2023 Oct 4;31(2):191–202. doi: 10.1093/eurjpc/zwad317 (PMC10809172; doi:10.1093/eurjpc/zwad317)
Supplement: zwad317_Supplementary_Data [file zwad317_supplementary_data.zip › Supplementary_text_11102023.pdf]

Text S1: Population quality control

REGICOR: Dataset with all participants in the 2005 cohort of the REGICOR study was used (n=6,352). We excluded participants with energy intake under and over reporters (women with <500 or > 3,500 kcal and men with <800 or >4,000 kcal) (Willett, 2012). A total of 646 participants had information of methylation sequenced using the Infinium HumanMethylation450, and 595 participants had information of methylation sequenced using the IlluminaHumanMethylationEPIC. This last sample was a case-control study of coronary heart disease, of which we selected only the controls (n=298) (Fernández-Sanlés *et al.*, 2021). Finally, 573 participants had information on phenotypic data (including diet and covariates) for the 450k array, and 269 for the EPIC array.

FOS: Information on diet was collected in examination 7 and methylation data in examination 8. From the total number of participants (n=2,542), 2,430 had a valid food frequency questionnaire [FFQ] (validity marker for FFQ based number of blanks and plausible energy intake). Following instructions from Framingham, for men, less than 13 blanks on FFQ and energy intake between 600-4,199 kcal were allowed; for women, less than 13 blanks and energy intake between 600-3,999 kcal were allowed. Finally, a total of 1,843 participants with information for phenotypic data (including diet and covariates) and methylation were included.

WHI: We used a subset of the original data with only the baseline visit, when DNA methylation was assessed (n=117,469). We had phenotypic data for 1,863 participants. 1,856 participants had information for both phenotypic and food-nutrients data. We excluded participants having over or under reporting energy intake (above 5,000 kcal and below 600 kcal), keeping 1,777 participants with plausible energy intake. Besides, we followed instructions from WHI dbGaP Cohort Phenotype Data Release, Data Preparation Guide, Updated May 2018 (for phs000200.v12), WHI Outcomes Through March 31, 2018: “Consider excluding all nutrient measures for participants with total energy (kcal) less than 600 or greater than 5000 as these energy intake estimates suggest that participants did not complete the FFQ in a reasonable manner.” 17 participants with total energy > 4,000 kcal were excluded. Finally, 1,736 participants had information on phenotypic data (including diet and covariates) and methylation.

Airwave: We used a subsample of the original dataset of 1,130 participants. We excluded 75 participants with no information of diet and methylation and excluded non-white participants (n=29). Besides, and as stated by Imperial College, multivariate outlier identification was performed using the *pcout()* function of the *mvoutlier* R package (Filzmoser and Gschwandtner, 2021), leaving out 182 participants as potential outliers. All participants met the energy intake inclusion criteria (>600 kcal and <5,000 kcal average daily energy intake).

Fernández-Sanlés,A. et al. (2021) DNA methylation biomarkers of myocardial infarction and cardiovascular disease. Clin. Epigenetics, 13, 1–11.

Text S2: Computation of diet quality scores

The MMDS (Ma *et al.*, 2020) includes nine components: vegetables, fruits, nuts, legumes, whole grains, fish, red meat, ratio of monounsaturated fatty acids to saturated fatty acids, and alcohol. Intake of each food component was categorized into sex- and cohort-specific quartiles, and scores of 0, 1, 2 and 3 were assigned to the lowest to the highest quartile for all components except red meat (3, 2, 1 and 0 from the lowest to the highest quartile of intake). For alcohol (scored 0/1), a score of 1 was given if consumption was ≥ 10 grams/day and ≤ 25 grams/day for men or ≥ 5 grams/day and ≤ 15 grams/day for women and 0 for all other values. The total score ranges 0-25, with higher values reflecting higher adherence to a traditional Mediterranean diet.

The DASH diet score (Fung *et al.*, 2008) includes eight components: fruits, vegetables, nuts and legumes, low-fat dairy products, and whole grains (positive), sodium, sweetened beverages, and red and processed meats (negative). These were categorized into sex- and cohort-specific quintiles, and scores of 1 to 5 were attributed to the lowest to the highest quintiles of the positive components and to the highest to the lowest quintiles for negative components. The total score ranges 8-40, with higher values reflecting higher adherence to a DASH diet.

The HPDI (Shan *et al.*, 2020) includes 18 components: seven healthy plant foods (whole grains, fruits, vegetables, nuts, legumes, vegetable oils, and tea and coffee), five less healthy plant foods (fruit juices, refined grains, potatoes, sugar-sweetened beverages, and sweets and desserts) and six animal foods (animal fat including butter or lard, dairy, eggs, fish and seafood, meat, and miscellaneous animal-based foods). Intake was categorized into sex- and cohort-specific quintiles, and each quintile was assigned a score between 1 and 5 to the lowest to the highest quintiles of the healthy plant foods and to the highest to the lowest quintiles of the unhealthy plant foods and animal foods. By summing scores of 18 food groups, the HPDI ranges 18-90, with a higher score indicating a healthier plant-based diet.

Fung,T.T. et al. (2008) Adherence to a DASH-style diet and risk of coronary heart disease and stroke in women. Arch. Intern. Med., 168, 713–720.

Ma,J. et al. (2020) Whole Blood DNA Methylation Signatures of Diet Are Associated With Cardiovascular Disease Risk Factors and All-Cause Mortality. Circ. Genomic Precis. Med., 13, e002766.

Shan,Z. et al. (2020) Association Between Healthy Eating Patterns and Risk of Cardiovascular Disease. JAMA Intern. Med., 180, 1090–1100.

Text S3: DNA methylation pre-processing for the Airwave study

Mean intensities from non-control beads were rearranged into two matrices with intensity data for each of the alleles at a specific genomic location. One matrix corresponded to absence of DNA methylation (A) and the other matrix corresponded to presence of DNA methylation (B).

Censoring values below detection limits:

Detection thresholds were estimated from the 600 'negative' control beads as follows:

$2\overline{x}_G + Z_\alpha\sqrt{2s_G}$ Infinium I measured in green

$2\overline{x}_R + Z_\alpha\sqrt{2s_R}$ Infinium I measured in red

$\overline{x}_G + \overline{x}_R + Z_\alpha\sqrt{S^2{}_G + S^2{}_R}$ Infinium II measured in green

Where $\overline{x}_G$ and $\overline{x}_R$ are the means, s_G and S_R are the standard deviations of the background noise in each colour separately obtained from the 'negative' control beads and $Z_\alpha = \Phi^{-1}(1-\alpha)$.

Elements of A and B were censored if the total intensity I=A+B was below these thresholds.

The control for the background noise and since the 'negative' control beads should not hybridise, background subtraction was done in addition to censoring, by subtracting mean intensities of 'negative' beads from A and B, depending on the colour each is measured in, and censoring negative values.

Dye bias which refers to the differences in fluorescence between the two colours was another source of bias in the measurements. This bias wouldn't affect Infinium I assay since the two bead types fluoresce in the same colour. But in Infinium II assay, since the same bead was used fluorescing in two colours, DNA methylation measurement is affected by this bias. For this purpose, normalisation control beads, which consists of 85 pairs of intensities in two green and red colours were used. The dye bias correction constant was computed as follows and then applied to the A and B matrices restricted to the Infinium II assays.

$k/\overline{r}$ for red intensities

$k/\overline{\overline{g}}$ for green intensities

With $\overline{g} = \Sigma_i g_i \,/\, 85$ and $\overline{r} = \overline{\Sigma}_i r_i \,/\, 85$, the mean intensities from normalisation control beads in green and red respectively and $k = (\overline{g} + \overline{r}) \,/\, 2$.

Finally, DNA methylation levels (β-values) were calculated as B (methylated) over total (A+B).

We transformed them into M-values, using the following formula:

$$M_{value} = log_2 \frac{(\beta)}{(1-\beta)}$$

Text S4: Mendelian randomization assumptions and sensitivity analyses

Three key assumptions must be met in MR to have valid conclusions: 1) the genetic instruments used are robustly related to the exposure, 2) no confounding of the genetic instrument-outcome associations is present, and 3) the genetic instruments are exclusively linked to the outcome by the exposure of interest (Burgess *et al.*, 2019).

Regarding the first assumption, we calculated the robustness of our genetic instruments as the mean F-statistic (average beta2/standard error2 across all SNPs; F-statistics < 10 indicate the genetic instrument is weak) (Burgess *et al.*, 2019). In relation to the second assumption, we minimized the potential confounding of the genetic instrument-outcome association due to population structure by: 1) picking our genetic instruments from GWASs performed in populations of European ancestry; 2) using GWASs adjusted for genetic principal components (Wang *et al.*, 2015). Finally, whether a genetic instrument is linked to other risk factors for the outcome different than the exposure of interest (horizontal pleiotropy), the third MR assumption is violated (Davey Smith and Hemani, 2014). We assessed whether there was evidence of horizontal pleiotropy by procedures based on sensitivity MR methods (MR-Egger, weighted median, and weighted mode) (Hemani *et al.*, 2018). It can be detected if: 1) the MR-Egger method shows a non-zero intercept; 2) there is lack of concordance among MR estimates in inverse variance weighted and the alternative methods; and 3) between-SNP heterogeneity is observed according to the Cochran’s Q and the Rücker’s Q (Hemani *et al.*, 2018). The third condition to detect horizontal pleiotropy will not be taken into account for BMI since between-SNP heterogeneity in this trait can be explained by biological reasons beyond the lack of methodological validity of its genetic instruments (variants affect BMI by a plethora of physiological mechanisms and have diverse effects in different life stages).

Burgess,S. *et al.* (2019) Guidelines for performing Mendelian randomization investigations. *Wellcome open Res.*, **4**, 186.

Davey Smith,G. and Hemani,G. (2014) Mendelian randomization: genetic anchors for causal inference in epidemiological studies. *Hum. Mol. Genet.*, **23**, R89-98.

Filzmoser,P. and Gschwandtner,M. (2021) mvoutlier: Multivariate Outlier Detection Based on Robust Methods.

Hemani,G. *et al.* (2018) Evaluating the potential role of pleiotropy in Mendelian randomization studies. *Hum. Mol. Genet.*, **27**, R195–R208.

Wang,C. *et al.* (2015) Improved ancestry estimation for both genotyping and sequencing data using projection procrustes analysis and genotype imputation. *Am. J. Hum. Genet.*, **96**, 926–937.
